# The Therapeutic Potential of MicroRNAs Delivered By Mesenchymal Stem Cells in Parkinson’s Disease, Alzheimer’s Disease, and Stroke: A Systematic Review of Preclinical Studies

**DOI:** 10.1007/s12035-026-06247-3

**Published:** 2026-10-03

**Authors:** Dominika Przywara, Alicja Petniak, Paulina Gil-Kulik

**Affiliations:** 1 Department of Clinical Genetics, Medical University of Lublin, 11 Radziwillowska Str, Lublin, 20-080 Poland; 2 Doctoral School, Medical University of Lublin, Lublin, 20-093 Poland

**Keywords:** MicroRNAs, Mesenchymal stem cells, Parkinson disease, Alzheimer disease, Stroke

## Abstract

Population aging has led to a substantial increase in the prevalence of neurological disorders, including Parkinson’s disease (PD), Alzheimer’s disease (AD), and stroke. As current therapeutic strategies are largely limited to symptomatic management, the identification of disease-modifying treatments remains a major unmet clinical need. Mesenchymal stem cells (MSCs) and MSCs-derived microRNAs (miRNAs) have emerged as promising therapeutic candidates for these conditions. A systematic literature search was conducted in the PubMed and Scopus databases in accordance with the PRISMA guidelines. The aim was to synthesize the available evidence regarding the effects of MSCs-derived miRNAs on cellular processes, including apoptosis, proliferation, inflammation, oxidative stress, and motor function in experimental models of PD, AD, and stroke. Risk of bias was assessed using the SYRCLE Risk of Bias tool for animal intervention studies and the modified OHAT Risk of Bias Rating Tool (mOHAT). A total of 37 preclinical studies were included in the review: 9 focused on PD, 10 on AD, and 18 on stroke. The synthesized evidence indicates that specific MSCs-derived miRNAs, particularly miR-133b, miR-146a, miR-17–92, and miR-223, exert significant neuroprotective effects. These miRNAs actively modulate gene expression, attenuate neuroinflammation and apoptosis, and reduce the accumulation of disease-specific pathological markers, including α-synuclein and amyloid-β (Aβ). Furthermore, targeted delivery of these miRNAs was associated with improved motor and cognitive outcomes across the evaluated animal models. MSCs-derived miRNAs demonstrate considerable therapeutic potential for the treatment of neurodegenerative diseases and stroke through the modulation of multiple pathological pathways. However, as the current evidence is derived exclusively from in vitro and animal studies, future research should focus on the development of safe, standardized, and reproducible protocols to facilitate the translation of these findings into human clinical trials. The review was registered in the Open Science Framework (OSF): https://osf.io/cxt9b.

**Graphical Abstract:**

**Figure:**
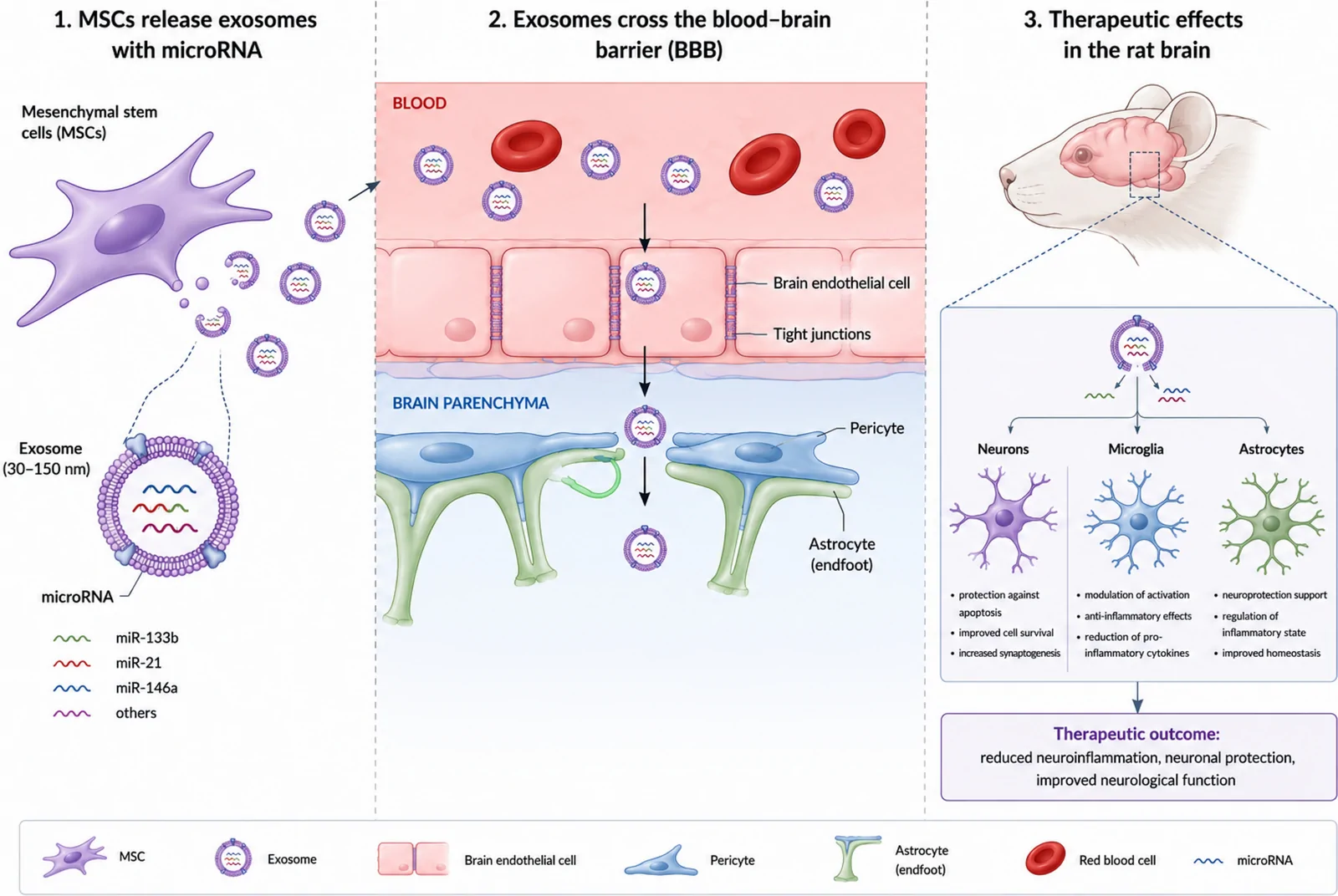

**Supplementary Information:**

The online version contains supplementary material available at https://doi.org/10.1007/s12035-026-06247-3.

## Introduction

The ongoing phenomenon of an ageing society is causing neurological disorders, for which advanced age constitutes a risk factor, to become an increasing concern. These conditions include, among others, neurodegenerative diseases and stroke [1].

Neurodegenerative diseases are conditions characterized by the progressive dysfunction of nerve cells. This process is primarily associated with cellular aging, which advances with the patient’s age [2, 3]. In turn, stroke is caused by vascular factors. Although it is not classified as a neurodegenerative disease, it leads to the degeneration of brain cells. These are severe disorders that frequently impair independent functioning [4, 5].

Parkinson’s disease (PD) and Alzheimer’s disease (AD) are the two most prevalent neurodegenerative disorders [6]. The pathomechanism of PD is generally associated with the loss of dopaminergic neurons and the formation of α-synuclein deposits (α-syn). The most characteristic symptom of PD is resting tremor; however, PD is a more complex disorder. In addition to tremor, such symptoms as bradykinesia, cognitive impairment, and depression are also observed [7, 8]. Moreover, gastrointestinal, cardiovascular, urogenital, and sexual dysfunctions are commonly reported [9].

The cause of AD is the amyloid precursor protein, which generates Aß peptides. These, in turn, form Aß plaques (Aß) in the brain, which are responsible for the development of neuroinflammation [10]. The progression of AD is also influenced by the formation of neurofibrillary tangles, which are associated with abnormal tau protein accumulation and synaptic loss [11]. The primary consequence of these processes is progressive dementia, which is related to the loss of cognitive functions. Patients with AD typically experience memory loss and cognitive impairment [10].

Stroke is caused by ischemia in a region of the brain, which leads to hypoxia, and may consequently result in the death of brain cells. The symptoms vary depending on the size and the location of the stroke [4].

Currently, the treatment of PD, AD, and stroke focuses on symptom management [1]. However, the pathomechanisms of these disorders are highly complex. Therefore, therapy should address multiple aspects. One of the proposed treatments is the application of mesenchymal stem cells (MSCs). MSCs are cells that, first, can differentiate into nerve cells and replace those that are damaged [12]; second, they secrete exosomes (MSCs-Exo), which serve as carriers of paracrine-active substances [13]. Among these factors are microRNAs (miRs) [1].

miRs are non-coding RNAs that regulate gene expression. miRs bind to mRNA and inhibit protein translation. In this way, miRs influence many cellular processes, which can be leveraged in therapy as modulatory factor targeting the pathomechanisms of disorders [14]. Understanding the role of miRs in PD, AD, and stroke may enable a more causal approach to treatment.

The aim of this review is to synthesize current knowledge on the effects of miRs derived from MSCs on apoptosis, proliferation, inflammation, oxidative stress, α-syn and Aβ levels, as well as cognitive functions in models of PD, AD, and stroke.

## Methods

### Eligibility Criteria and Sources

This review was conducted in accordance with PRISMA guidelines (https://www.prisma-statement.org/) (Supplementary Material 1). Publications focusing on the potential therapeutic effects of miRs derived from MSCs on PD, AD, and stroke were included in the study. No time restrictions were applied during the literature search. Selected studies were performed on animal models and cell lines.

There were also no restrictions on the type of material, participants’ age, sex, or disease stage. In animal studies, models were used in which the disease was either induced during the experiment or specifically designed for this purpose. There were also no restrictions on the type of cell line.

For this review, we included publications that assessed the role of miRs derived from MSCs in the pathomechanisms of PD, AD, and stroke, whose findings may have therapeutic applications. Other types of non-coding RNAs were excluded from this analysis. We also excluded studies that assessed only miR expression without evaluating their functional effects.

In order to identify publications, we searched the PubMed and Scopus databases. The most recent search was conducted on 10 June 2026. The publications included in the review were divided into three groups, depending on the type of disorder. The analysis of these publications was performed within these groups.

This systematic review was registered on 27 July 2025, in the Open Science Framework (https://osf.io/cxt9b).

### Search Strategy and Selection Process

For Scopus, publications were searched using the title, abstract, and keywords. For PubMed, we applied Medical Subject Headings (MeSH) terms. The following keywords were employed in the search: *neurodegenerative diseases*, *Parkinson disease*, *Alzheimer disease*, *stroke*, *microRNAs*, and *mesenchymal stem cells*. First, we excluded duplicate records and review articles. Of the remaining articles, we selected those that matched the topic of this review.

The selected publications were collected and then independently assessed by two researchers. There was no disagreement between the researchers during the evaluation.

### Study Risk of Bias Assessment

The review included preclinical studies conducted in both animal models and cell culture systems. To assess the risk of bias, SYRCLE’s Risk of Bias tool was used for animal studies, whereas the modified OHAT Risk of Bias Rating Tool (mOHAT) was applied to studies involving cell cultures. For animal studies, the standard SYRCLE questionnaire was used to evaluate methodological domains including random sequence generation, allocation concealment, and blinding of investigators and outcome assessors. Each item was rated as “yes,” “no,” or “unclear.” The “unclear” category was assigned when insufficient information was provided in the publication to permit a definitive judgment regarding a given domain [15].

For studies based on cell culture models, the OHAT Risk of Bias Rating Tool was adapted to better reflect the methodological characteristics of in vitro research. Specifically, Question 3 of the original tool (“Did selection of study participants result in appropriate comparison groups?”) was modified to: “Did the selection and allocation of cell cultures result in comparable experimental and control groups prior to treatment?”. Furthermore, because the reliability of cell culture experiments depends on maintaining identical culture conditions for both experimental and control groups before intervention, Questions 4 and 5 of the original OHAT tool were combined and reformulated as: “Were the cell cultures maintained under identical and stable conditions prior to the intervention?”. All remaining items were retained without modification (Supplementary Material 2).

For the mOHAT assessment, each domain was rated as follows: Definitely Low Risk of Bias (+ +), Probably Low Risk of Bias (+), Probably High Risk of Bias (− NR), or Definitely High Risk of Bias (−). An additional “unclear” category was introduced when the available information was insufficient to determine the risk of bias for a particular domain.

### Effect Measures

Effect measures consisted of findings from various studies that assessed the functioning of nerve cells in PD, AD, and stroke. We considered, among others, gene expression, markers of neurodegenerative disorders, inflammatory factors, ROS levels, apoptosis index, proliferation rate, as well as behavioral test results in animal models. The data were compared with those from control and sham groups.

### Certainty Assessment

Although the findings of the included studies are promising, the overall certainty of the evidence is considered low to moderate. While individual studies demonstrated internal consistency and frequently corroborated their findings using multiple rigorous methods (e.g., qPCR in conjunction with Western blotting or behavioral assays), the overall certainty is limited by several methodological constraints. In particular, the in vivo studies were based on small animal cohorts and often lacked clear reporting of allocation concealment, randomization of animal housing, and blinding of investigators or outcome assessors. Furthermore, substantial heterogeneity in experimental models, types of interventions (unmodified MSCs versus modified exosomes), and outcome measures contributes to limited confidence in the translational relevance of the findings. Therefore, a cautious interpretation of the current preclinical evidence is warranted until these findings are validated in larger, highly standardized studies.

## Results

### Study Selection

**Fig. 1:**
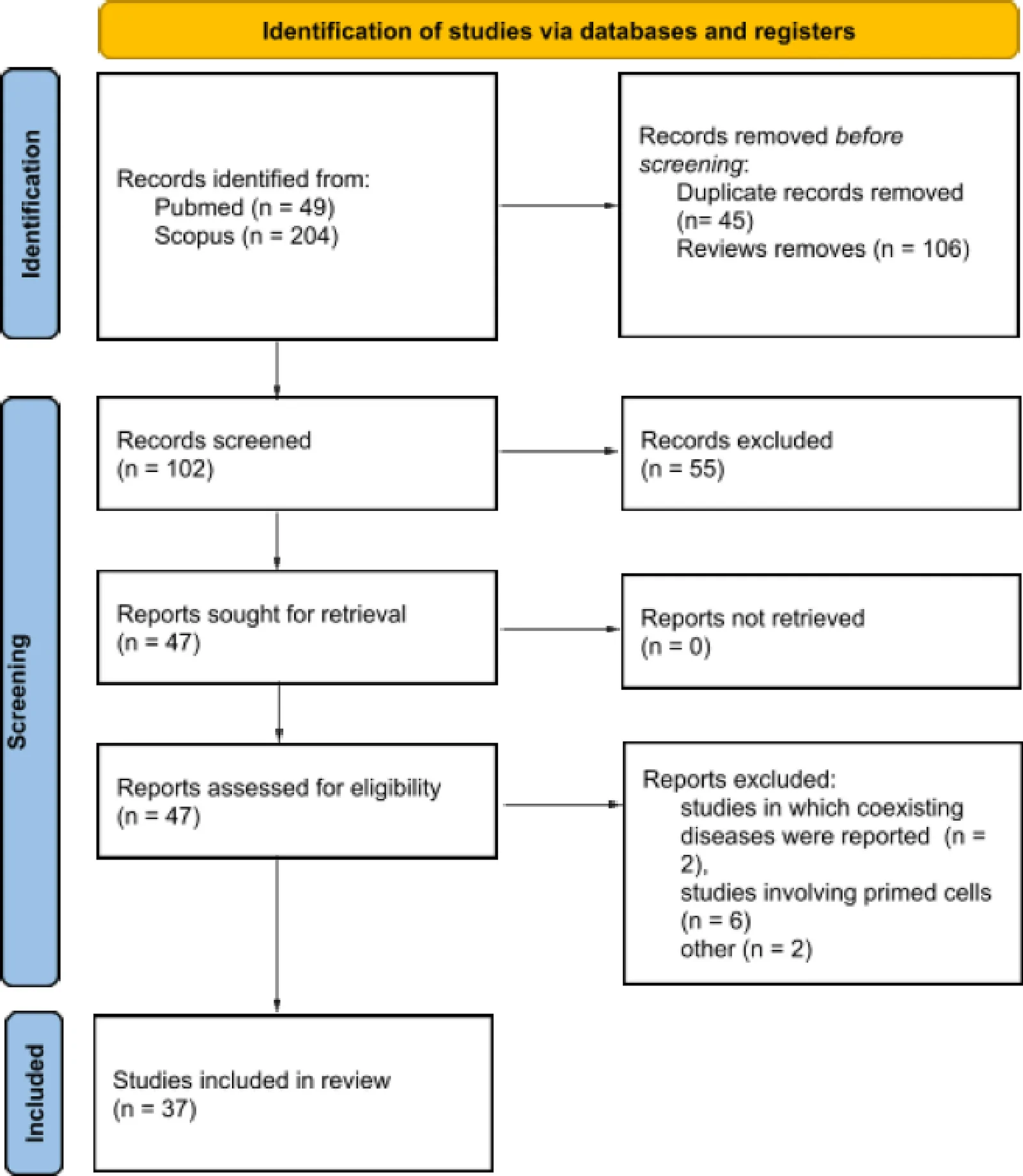
Flow diagram illustrating the publication selection process

Two databases were searched, and a total of 253 publications were collected. After removing reviews and duplicates, 102 research articles remained. Next, we excluded publications that did not investigate the combined therapeutic approach involving miRs delivered by MSCs. As a result, 47 studies met the criteria for inclusion. In the final stage of selection, studies involving models with disorders other than PD, AD, or stroke, or those using primed MSCs, were excluded. Consequently, 37 publications were included in this review (Fig. 1).

This systematic review focuses on the actions of miRs derived from MSCs, thus, studies that do not include both of these elements were not taken into account [16]. Furthermore, to minimize the risk of confounding factors influencing the effects of miRs, we excluded studies involving models with comorbid diseases such as diabetes [17]. Moreover, we discarded publications in which MSCs were previously primed or modulated by external factors other than the evaluated miRs [18–20]. Studies in which MSCs-derived exosomes (MSCs-Exo) were used to deliver not miRs but only their inhibitors were not considered [21].

### Study Characteristics

This review comprises 9 publications on PD models, 10 on AD models, and 18 on stroke. The studies include material in the form of cell lines and animal models, in which PD, AD, and stroke were induced using various factors. The number of animals utilized in the studies ranged from 6 to 15 per group for PD, 3 to 10 for AD and 3 to 42 for stroke. This review concentrated on the specific effect of miRs derived from MSCs or MSCs-Exo on disease models, rather than the general influence of MSCs on disease. The function of miRs was investigated using PCR, ELISA test, flow cytometry, Western blot, immunohistochemistry, biochemical tests, various staining methods, and behavioral tests (Tables 1, 2, and 3).

**Table 1:** Characteristics of studies in terms of materials used, types of miRs, intervention methods, and techniques applied to assess miRs functions in PD models

Author, date	Material	PD induction method	Number of animals per group	Type of miR	Type of intervention	Type of MSCs modification	Methods used to evaluate miR function	References
Geng, 2023	Sprague–Daw rats	6-OHDA	10–15	miR-23b-3p	MSCs-Exo application	transfection of the miR-23b-3p inhibitor	qPCR, Elisa test, Western blot, immunohistochemistry,	[22]
	MN9D cell line		-		culture with MSCs-Exo			
Mohamed, 2023	Albino rats	rotenone	10	miR-34b	MSCs-Exo application	unmodified MSCs-Exo	qPCR, Elisa test	[23]
He, 2023	C57BL/6 mice	MPTP	10	miR-100-5p	MSCs application	unmodified MSCs	qPCR, Western blot, behavioral tests	[24]
	MN9D cell line	MPP +	-		culture with MSCs	transfection of the miR-100-5p inhibitor and mimic		
Ma, 2022	PD mouse model	6-OHDA	8	miR-181a-2-3p	MSCs-Exo application	transfection of the miR-181a–2–3p precursor	qPCR, Western blot, flow cytometry, fluorescent staining, immunohistochemical staining, MTT assay, behavioral tests	[25]
	SH-SY5Y cell line		-		miR transduction	-		
Mahdy, 2023	Swiss mice	rotenone	8	miR-155	MSCs application	unmodified MSCs and preconditioned MSCs	qPCR, immunohistochemical study, histomorphometric study	[26]
Li, 2021	C57BL/6 J mice	MPTP	8	miR-188-3p	MSCs-Exo application	transfection of the miR-188-3p mimic	qPCR, Elisa test, Western blot, flow cytometry, immunohistochemical study, immunofluorescence analysis, FISH, 5-Ethynyl-2′-deoxyuridine (EdU) assay, behavioral tests	[27]
	MN9D cell line	MPP +	-		miR transfection	-		
Yang, 2024	C57BL/J mice	exposure to MnCl2	6	miR-494–3p	MSCs-Exo application	transfection of the miR-494–3p mimic	qPCR	[28]
	BV2 cell line		-		miR transfection	-		
Forouzandeh, 2022	Wistar rats	6-OHDA	8	miR-149-5p	MSCs application	unmodified MSCs	qPCR, behavioral tests	[29]
Jiang, 2024	C57BL/6 J mice	6-OHDA	6	miR-133b	MSCs-Exo application	loading miR-133b mimic into exosomes via incubation	qPCR, Western blot, immunohistochemical study, behavioral tests	[30]

**Table 2:** Characteristics of studies in terms of materials used, types of miRs, intervention methods, and techniques applied to assess miRs functions in AD models

Author, date	Material	AD induction method	Number of animals per group	Type of miR	Type of intervention	Type of MSCs modification	Methods used to evaluate miR function	References
Nakano, 2020	APP/PS1 mice	the AD model was used	3–5	miR-146a	MSCs application	unmodified MSCs	qPCR	[31]
	astrocytes	-	-		MSCs culture and culture with isolated MSCs-Exo	transfection of the miR-146a mimic		
Liu, 2015	APP/PS1 mice	the AD model was used	10	miR-937	MSCs application	as-miR-937 transduction	qPCR, Western blot, immunohistochemical study, behavioral tests	[32]
Sha, 2021	SD rats	the AD model was used	6	miR-29c-3p	MSCs-Exo application	unmodified MSCs	qPCR, Elisa test, Western blot, flow cytometry, MTT assay	[33]
	hippocampal neuron	treatment of Aβ1–42	-		treated with MSCs-Exo	transfection of the miR-29c-3p inhibitor		
Zhai, 2021	APP/PS1 mice	the AD model was used	10	miR-22	MSCs-Exo application	transfection of the miR-22 mimic	qPCR, Elisa test, Western blot, flow cytometry, immunofluorescence staining, behavioral tests	[34]
Wei, 2020	SH-SY5Y cell line	amyloid β protein fragment	-	miR-223	culture with MSCs and MSCs-Exo	inhibition of miR-223 in MSCs using an antagomir and its overexpression using an agomir	qPCR, Western blot	[35]
Chen, 2023	SH-SY5Y cell line	Aβ1‐40	-	miR‐211‐5p	culture with MSCs-Exo	transfection of the miR‐211‐5p mimic and inhibitor	qPCR, Elisa test, Western blot, flow cytometry, transwell assay	[36]
Jahangard, 2020	Wistar rats	Aβ1–42	6	miR-29	MSCs application	using recombinant miR-29 expression vectors	qPCR, behavioral tests	[37]
Lin, 2024	C57BL/6 J mice	streptozotocin	10	miR-223-3p	MSCs-Exo application	using a miR-223-3p inhibitor in MSCs	Western blot	[38]
Zhang, 2025	Sprague–Dawley rats	Aβ1–42	8	miR-214-3p	MSCs-Exo application	transfection of the miR-214-3p	TUNEL staining, qPCR, Western blot, behavioral tests	[39]
Zhang, 2025	HT22 cell line	Aβ1–42	-	miR-22-3p	MSCs-Exo application	transfection of the miR-22-3p mimic and inhibitor	CCK-8 assay, Oxidative stress assay, Flow cytometry, Western blot, RT-qPCR	[40]

**Table 3:** Characteristics of studies in terms of materials used, types of miRs, intervention methods, and techniques applied to assess miRs functions in stroke models

Author, date	Material	Stroke induction method	Number of animals per grouph na grupę	Type of miR	Type of intervention	Type of MSCs modification	Methods used to evaluate miR function	References
Xin, 2021	Wistar rats	middle cerebral artery occlusion (MCAO)	10	miR-17–92	MSCs-Exo application	pre-miRNA-17–92 transduction	histochemistry, immunohistochemistry, electrophysiological measurement, behavioral tests	[41]
Xin, 2017	Wistar rats	MCAO	6	miR-133b	MSCs-Exo application	pre-miR-133b and miR-133b inhibitor transduction	Western blot, histopathology, immunohistochemistry, behavioral tests	[42]
Xie, 2023	C57BL/6 J mice	MCAO	5	miR-206	MSCs-Exo application	transfection of the miR-206 mimic	Western blot, MTT assay, TUNEL assay, ROS assay, dual luciferase assay	[43]
	BV-2 cell line	oxygen–glucose deprivation (OGD)	-		culture with MSCs-Exo			
Zhang, 2021	C57BL/6 mice	MCAO	6–12	miR-146a-5p	MSCs-Exo application	unmodified MSCs	Western blot, 2,3,5-triphenyltetrazolium chloride (TTC) staining	[44]
	BV-2 cell line	OGD	-		culture with MSCs-Exo	miR-146a-5p inhibitor transduction		
Hu, 2022	ICR mice	MCAO	3–12	miR-21-5p	MSCs-Exo application	transfection of the miR-21-5p	qPCR, Western blot	[45]
Li, 2019	Sprague–Dawley rats	MCAO	42	miR-21-3p	MSCs application	unmodified MSCs	qPCR, Elisa test, TUNEL staining, TTC staining	[46]
	SH-SY5Y cell line	incubating in hypoxic conditions	-		culture with MSCs-Exo	unmodified MSCs, transfection of the miR-21-3p mimic and inhibitor to SH-SY5Y		
Kuang, 2020	C57BL/6 mice	MCAO	6–10	miR-25-3p	MSCs-Exo application	unmodified MSCs and transfection of the miR-25-3p inhibitor	qPCR, Western blot, MTT assay, fluorescence microscopy	[47]
	cortical neurons	OGD	-		culture with MSCs-Exo			
Li, 2022	Sprague Dawley rats	MCAO	3–6	miR-150-5p	MSCs-Exo application	transfection of the miR-150-5p mimic	qPCR, Western blot, Elista test, TUNEL staining, hematoxylin–eosin (HE) staining, dual luciferase reporter gene assay	[48]
Xin, 2017	Wistar rats	MCAO	8	miR-17–92	MSCs-Exo application	transfection of the miR-17–92	histochemistry, immunohistochemistry, Golgi-Cox staining, behavioral tests	[49]
Xin, 2013	Wistar rats	MCAO	6	miR-133b	MSCs application	miR-133b mimic and inhibitor transduction	qPCR, biotinylated dextran amine label, histochemistry, immunostaining, behavioral tests	[50]
Yi, 2024	HMC3 cell line	OGD	-	miR-148b-3p	culture with MSCs-Exo	transfection of the miR-148b-3p mimics and inhibitor to MSCs	qPCR, Elisa test, MTT assay, clonal formation assay, luciferase reporter assays	[51]
Duan, 2020	Sprague–Dawley rats	injection of collagenase type IV	6	miR-146a-5p	MSCs-Exo application	miR-146a-5p transduction	qPCR, Western blot, FJB and TUNEL staining, immunofluorescence assay, dual-luciferase assay, behavioral test	[52]
Liang, 2024	BV2 cell line	stimulating with hemoglobin	-	miR-197-3p	culture with MSCs-Exo	unmodified MSCs and transfection of the miR-197-3p mimic and inhibitor	qPCR, dual-luciferase assay	[53]
Bao, 2024	Sprague–Dawley rats	MCAO	3–12	miR-486	MSCs-Exo application	transfection of the miR-486 mimic and inhibitor	qPCR, Western blot, immunofluorescence staining, scratch wound healing assay, transwell migration assay, tube formation assay	[54]
Shen, 2026	C57BL/6 mice	MCAO	10–15	miR-664a-5p	MSCs-Exo application	unmodified MSCs	Dual-luciferase reporter assay, TTC, HE, Nissl staining, Western blot, behavioral test,	[55]
	BV-2 cell line	no intervention	-		culture with MSCs-Exo	transfection of the miR-664a-5p mimic		
Yang, 2026	astrocytes and mouse brain microvascular endothelial cells	OGD	-	miR-21a-5p	culture with MSCs-Exo	unmodified MSCs, transfection of the miR-21a-5p mimic and inhibitor	HE and TUNEL staining, qRT-PCR, Western blot, ELISA, MMP Activity Assay Kit, BCA Protein Assay Kit	[56]
	C57BL/6 J mice	MCAO	15–30		MSCs-Exo application			
Zhang, 2026	Primary mouse neuronal cells	OGD	-	miR-137	culture with MSCs-Exo	loading of a miR-137 inhibitor into MSCs-Exo	qPCR, Dual-Luciferase Reporter Assay, Western blot, Nanoparticle Tracking Analysis, Transmission Electron Microscopy, and Near-infrared Fluorescence Imaging	[57]

### PRISMA 2020 Flow Diagram for New Systematic Reviews Which Included Searches of Databases and Registers Only

#### Risk of Bias in Studies

Risk of bias was assessed using the SYRCLE Risk of Bias tool (Table 4) and the modified OHAT Risk of Bias Rating Tool (mOHAT; Table 5). For the SYRCLE assessment, each domain was rated as “yes,” “no,” or “unclear.” The “unclear” rating was most frequently assigned to Questions 3 and 4, which addressed allocation concealment and the random housing of animals, respectively. Among the studies included in the review, the lowest score observed was six positive (“yes”) responses.

**Table 4:** Risk of bias assessment of the included publication using the SYRCLE’s risk of bias tool for animal studies

Studies	Questions									
	1	2	3	4	5	6	7	8	9	10
Geng, 2023 [22]	yes	yes	yes	unclear	unclear	yes	yes	yes	yes	yes
Mohamed, 2023 [23]	unclear	yes	unclear	unclear	unclear	yes	yes	yes	yes	yes
He, 2023 [24]	yes	yes	yes	unclear	yes	yes	yes	yes	yes	yes
Ma, 2022 [25]	yes	unclear	unclear	unclear	yes	yes	yes	yes	yes	yes
Mahdy, 2023 [26]	yes	unclear	unclear	unclear	yes	yes	yes	yes	yes	yes
Li, 2021 [27]	unclear	yes	unclear	unclear	yes	yes	yes	yes	yes	yes
Yang, 2024 [28]	yes	yes	unclear	unclear	unclear	yes	yes	yes	yes	yes
Forouzandeh, 2022 [29]	unclear	yes	unclear	unclear	yes	yes	yes	yes	yes	yes
Jiang, 2024 [30]	yes	yes	unclear	unclear	unclear	yes	yes	yes	yes	yes
Nakano, 2020 [31]	unclear	yes	unclear	unclear	unclear	yes	yes	yes	yes	yes
Liu, 2015 [32]	unclear	yes	unclear	unclear	yes	yes	yes	yes	yes	yes
Sha, 2021 [33]	yes	yes	unclear	unclear	yes	yes	yes	yes	yes	yes
Zhai, 2021 [34]	unclear	unclear	unclear	unclear	yes	yes	yes	yes	yes	yes
Jahangard, 2020 [37]	yes	yes	unclear	unclear	yes	yes	yes	yes	yes	yes
Lin, 2024 [38]	yes	yes	unclear	unclear	yes	yes	yes	yes	yes	yes
Zhang, 2025 [39]	yes	yes	unclear	unclear	yes	yes	yes	yes	yes	yes
Xin, 2021 [41]	unclear	unclear	unclear	unclear	yes	yes	yes	yes	yes	yes
Xin, 2017 [42]	yes	yes	unclear	unclear	yes	yes	yes	yes	yes	yes
Xie, 2023 [43]	unclear	yes	unclear	unclear	yes	yes	yes	yes	yes	yes
Zhang, 2021 [44]	yes	yes	unclear	unclear	yes	yes	yes	yes	yes	yes
Hu, 2022 [45]	unclear	yes	unclear	unclear	yes	yes	yes	yes	yes	yes
Li, 2019 [46]	yes	yes	unclear	unclear	unclear	yes	yes	yes	yes	yes
Kuang, 2020 [47]	yes	yes	unclear	unclear	yes	yes	yes	yes	yes	yes
Li, 2022 [48]	unclear	unclear	unclear	unclear	yes	yes	yes	yes	yes	yes
Xin, 2017 [49]	yes	unclear	unclear	unclear	yes	yes	yes	yes	yes	yes
Xin, 2013 [50]	unclear	unclear	unclear	unclear	unclear	yes	yes	yes	yes	yes
Duan, 2020 [52]	unclear	yes	unclear	unclear	yes	yes	yes	yes	yes	yes
Bao, 2024 [54]	unclear	yes	unclear	unclear	unclear	yes	yes	yes	yes	yes
Shen, 2026 [55]	yes	unclear	unclear	unclear	yes	yes	yes	yes	yes	yes
Yang, 2026 [56]	yes	yes	unclear	unclear	unclear	yes	yes	yes	yes	yes
Zhang, 2026 [57]	unclear	yes	unclear	unclear	yes	yes	yes	yes	yes	yes

**Table 5:** Risk of bias assessment of the included publication using the mOHAT risk of bias tool for cell culture

Studies	Questions									
	1	2	3	4	5	6	7	8	9	10
Geng, 2023 [22]	+	unclear	+ +	+ +	unclear	+ +	+ +	+ +	+ +	+ +
He, 2023 [24]	+	unclear	+ +	+ +	unclear	+ +	+ +	+ +	+ +	+ +
Ma, 2022 [25]	+	unclear	+ +	+ +	unclear	+ +	+ +	+ +	+ +	+ +
Li, 2021 [27]	+	unclear	+ +	+ +	unclear	+ +	+ +	+ +	+ +	+ +
Yang, 2024 [28]	unclear	unclear	unclear	unclear	unclear	+ +	+ +	+ +	+ +	+ +
Wei, 2020 [35]	+	unclear	+ +	+ +	unclear	+ +	+ +	+ +	+ +	+ +
Chen, 2023 [36]	+	unclear	+ +	+ +	unclear	+ +	+ +	+ +	+ +	+ +
Zhang, 2025 [40]	+	unclear	+ +	+ +	unclear	+ +	+ +	+ +	+ +	+ +
Zhang, 2021 [43]	+	unclear	+ +	+ +	unclear	+ +	+ +	+ +	+ +	+ +
Li, 2019 [46]	+	unclear	+ +	+ +	unclear	+ +	+ +	+ +	+ +	+ +
Kuang, 2020 [47]	unclear	unclear	+	+	unclear	+ +	+ +	+ +	+ +	+ +
Yi, 2024 [51]	unclear	unclear	+ +	+ +	unclear	+ +	+ +	+ +	+ +	+ +
Liang, 2024 [53]	+	unclear	+ +	+ +	unclear	+ +	+ +	+ +	+ +	+ +
Shen, 2026 [55]	+	unclear	+ +	+ +	unclear	+ +	+ +	+ +	+ +	+ +
Yang, 2026 [56]	+ +	unclear	unclear	unclear	unclear	+ +	+ +	+ +	+ +	+ +
Zhang, 2026 [57]	unclear	unclear	+ +	+ +	unclear	+ +	+ +	+ +	+ +	+ +

A considerable number of publications did not provide sufficient information regarding the blinding of investigators or outcome assessors, which may increase the risk of bias. Nevertheless, these studies were retained in the review due to the already limited number of eligible publications and because most outcomes were assessed using objective quantitative methods, such as quantitative polymerase chain reaction (qPCR) and enzyme-linked immunosorbent assay (ELISA), which are less susceptible to observer-related bias. Overall, while several studies exhibited methodological limitations related to reporting quality, the majority provided sufficient information to support the validity of their findings.

Although the SYRCLE Risk of Bias tool does not assess sample size adequacy, it should be noted that the studies included in this review were generally based on relatively small numbers of animals, ranging from 3 to 42 individuals per study. Small sample sizes increase the risk of false-positive findings and limit the robustness and reproducibility of the reported results, representing an important limitation of the available evidence. Nevertheless, these studies were included in the review due to the limited number of publications currently available on this topic and the exploratory nature of the field.

For the mOHAT assessment, the response options were: Definitely Low Risk of Bias (+ +), Probably Low Risk of Bias (+), Probably High Risk of Bias (− NR), and Definitely High Risk of Bias (−). For Question 1, studies were assigned a rating of Probably Low Risk of Bias (+) when randomization of cell line allocation was not explicitly reported, but the cell lines were obtained from an independent commercial supplier. This decision was based on the assumption that investigators were not involved in the generation or selection of the original cell lines and, therefore, their allocation of cells to experimental groups was unlikely to systematically influence study outcomes. For Question 3, a rating of Definitely Low Risk of Bias (+ +) was assigned when both the experimental and control groups were derived from the same cell line obtained from the same supplier. Under these conditions, the groups were considered comparable at baseline, minimizing the risk of bias resulting from intrinsic biological differences between cell populations.

Because the response categories “Probably High Risk of Bias” (− NR) and “Definitely High Risk of Bias” (−) require direct evidence indicating the presence of bias, an additional response category, “unclear,” was introduced for the purposes of this review. This category was assigned when a publication did not provide sufficient information to allow a reliable assessment of a given domain. For Question 1, an “unclear” rating was assigned when the source or supplier of the cell line was not reported. In such cases, it was not possible to determine whether the allocation of cell cultures could have introduced selection bias. The authors considered the remaining questions to be sufficiently specific and clearly formulated to permit an unambiguous assessment of risk of bias based on the information provided in the included studies.

## Results of Individual Studies

The compiled studies demonstrate the influence of certain types of miRs on cellular processes occurring in PD, AD, and stroke models. It has been observed that miRs can decrease the levels of α-syn and Aβ, influence apoptosis, inflammation, and oxidative stress. Furthermore, miRs modulate signaling pathways, protein levels, and gene expression.

It has been observed that in PD, miRs are able to modify the levels of proteins such as deglycase (DJ-1), parkin (PARKIN), NADPH oxidase 4 (Nox4), and tyrosine hydroxylase (TH).

In AD models, the influence of miRs on tumor necrosis factor receptor-associated factor 6 (*TRAF6*), nuclear factor NF-kappa-B (NF-κB), brain-derived neurotrophic factor (*BDNF*), POU domain, class 3, transcription factor 4 (*Brn4*), β-site amyloid precursor protein cleaving enzyme 1 (*BACE1*), phosphatase and tensin homolog (*PTEN*), neprilysin (*NEP*), Bcl2 interacting mediator of cell death (*BIM*), neuron navigator 3 (*NAV3*), and NLR family pyrin domain containing 3 (*NLRP3*) has been indicated.

In stroke models, a correlation between miRs and growth-associated protein 43 (GAP43), phosphorylated neurofilament heavy polypeptide (pNFH), Rab9 effector protein with kelch motifs (RABEPK), BCL2 interacting protein 3 (BNIP3), B-cell lymphoma-2-associated X protein (*BAX*), BCL2 apoptosis regulator (*BCL2*), toll-like receptor 5 (*TLR5*), connective tissue growth factor (CTGF), Delta-like ligand 4 (*DLL4*), neurogenic locus notch homolog protein 1 (*Notch1*), interleukin-1 receptor-associated kinase 1 (*IRAK1*), nuclear factor of activated T cells 5 (*NFAT5*), active phenotype of gasdermin D (*GSDMD-N*), and lipocalin 2 (*LCN2*) was observed.

In addition, it has been demonstrated that delivering certain miRs improves the results of behavioral tests (Tables 6, 7, and 8).

**Table 6:** Functions of examined miRs and their effects on PD models

Author, date	Type of miR	Functions of examined miR	References
Geng, 2023	miR-23b-3p	reduce α-syn levels, promotion of neuronal autophagy	[22]
Mohamed, 2023	miR-34b	reduce α-syn levels, increase DJ-1 and PARKIN levels	[23]
He, 2023	miR-100-5p	reduce oxidative stress, reduce Nox4 levels, increase *TH* expression, improve motor function	[24]
Ma, 2022	miR-181a-2-3p	reduce α-syn levels, increase viability, reduce apoptosis and oxidative stress, increase *TH* expression, improve motor function	[25]
Mahdy, 2023	miR-155	increase apoptosis	[26]
Li, 2021	miR-188-3p	reduce α-syn levels, inhibit autophagy, reduce pyroptosis, increase proliferation, increase *TH* expression, have anti-inflammatory effects, improve motor function	[27]
Yang, 2024	miR-494–3p	have anti-inflammatory effects	[28]
Forouzandeh, 2022	miR-149-5p	reduce α-syn levels, improve motor function	[29]
Jiang, 2024	miR-133b	increase *TH* expression, target the RhoA-ROCK pathway, improve motor function	[30]

**Table 7:** Functions of examined miRs and their effects on AD models

Author, date	Type of miR	Functions of examined miR	References
Nakano, 2020	miR-146a	decrease *TRAF6* and *NF-κB* expression in astrocytes, have anti-inflammatory effects	[31]
Liu, 2015	miR-937	inhibit *Brn4* translation, decrease *BDNF* expression, increase Aβ levels	[32]
Sha, 2021	miR-29c-3p	target the *BACE1*, activate Wnt/β-catenin pathway, reduce Aβ1–42 levels in neurons, reduce apoptosis	[33]
Zhai, 2021	miR-22	have anti-inflammatory effects, reduce pyroptosis, improve motor function	[34]
Wei, 2020	miR-223	target the *PTEN*, activate PI3K/Akt pathway, reduce neuronal apoptosis, have anti-inflammatory effects	[35]
Chen, 2023	miR‐211‐5p	targets the *NEP*, increase apoptosis and inflammation, reduce cell migration	[36]
Jahangard, 2020	miR-29	target the *BACE1*, *BIM* and *NAV3*, improve behavioral tests outcomes	[37]
Lin, 2024	miR-223-3p	target the *NLRP3* and *IL-1β*, have anti-inflammatory effects, reduce pyroptosis	[38]
Zhang, 2025	miR-214-3p	reduce oxidative stress, reduce neuronal apoptosis, improve behavioral tests outcomes	[39]
Zhang, 2025	miR-22-3p	Increases cell viability while reducing apoptosis and oxidative stress	[40]

**Table 8:** Functions of examined miRs and their effects on stroke models

Author, date	Type of miR	Functions of examined miR	References
Xin, 2021	miR-17–92	increase GAP43 expression, increase axonal density and plasticity, increase myelination, improve motor function	[41]
Xin, 2017	miR-133b	increase axonal density, enhance neurite remodeling, increase pNFH and synaptophysin expression, decrease RABEPK levels, improve motor function	[42]
Xie, 2023	miR-206	increase apoptosis, oxidative stress and inflammation	[43]
Zhang, 2021	miR-146a-5p	have anti-inflammatory effects, reduce the size of stroke lesion	[44]
Hu, 2022	miR-21-5p	have proangiogenic activity	[45]
Li, 2019	miR-21-3p	increase apoptosis and inflammation	[46]
Kuang, 2020	miR-25-3p	increase viability, inhibit autophagy, decrease p53 and BNIP3 levels	[47]
Li, 2022	miR-150-5p	inhibit necrosis and apoptosis, decrease *BAX*, *BCL2* expression, target the *TLR5*, have anti-inflammatory	[48]
Xin, 2017	miR-17–92	increase axonal and dendritic density and plasticity, increase synaptophysin expression, increase neurogenesis and oligodendrogenesis, reduce PTEN levels, improve motor function	[49]
Xin,2013	miR-133b	increase axonal density, reduce CTGF levels, improve motor function	[50]
Yi, 2024	miR-148b-3p	decrease viability, proliferation, and cell migration, have anti-inflammatory effects, decrease *DLL4* and *Notch1* expression	[51]
Duan, 2020	miR-146a-5p	reduce apoptosis and oxidative stress, have anti-inflammatory effects, decrease *IRAK1* and *NFAT5* expression, improve motor function	[52]
Liang, 2024	miR-197-3p	decrease *NLRP3, caspase1-p20* *GSDMD-N*, and *IL-18* expression	[53]
Bao, 2024	miR-486	have proangiogenic activity, reduce PTEN levels	[54]
Shen, 2026	miR-664a-5p	Modulates the AAK1/NF-κB signaling pathway, which may contribute to neuroprotective effects	[55]
Yang, 2026	miR-21a-5p	Preserves blood–brain barrier integrity, reduces infarct volume and cerebral edema, decreases intracerebral hemoglobin levels and erythrocyte accumulation, and attenuates necrosis, apoptosis, and inflammatory cell infiltration	[56]
Zhang, 2026	miR-137	Reduces Sirt1 expression, a gene implicated in neuroprotection, neuronal plasticity, modulation of neuroinflammation, and resistance to ischemic damage, including stroke	[57]

### Results of Syntheses

The included studies evaluated the effects of MSCs-based interventions-ranging from unmodified MSCs to isolated exosomes enriched with specific miRNAs-in models of PD, AD, and stroke. Although the findings of many studies were directly associated with the overexpression or inhibition of specific miRNA species, it should be emphasized that, in studies employing whole, unmodified MSCs, causality cannot be attributed exclusively to miRNAs. In such cases, the observed therapeutic effects are likely synergistic in nature, driven by miRNAs in conjunction with other biologically active components of the secretome, such as growth factors, cytokines, and lipids. This allows the evaluation of the function of various miRs in disease models.

The findings indicate that miRs modulate proliferation, neuronal apoptosis, and the development of inflammation and oxidative stress in various ways. The overexpression or inhibition of certain miRs was found to influence motor function in disease models.

The credibility of the assessment may be limited due to the small number of studies on this topic. Most studies investigated different types of miRs, making direct comparisons challenging. Furthermore, the exosomes used in therapy were derived from various types of MSCs.

Among the studied miRs, each type affected specific disease models. In most cases, overexpression of the assessed miRs had a therapeutic effect. The collected results indicate that miRs expression influences the course of PD, AD, and stroke. Nevertheless, the publication data were characterized by significant variability in experimental groups, intervention types, and particularly the types of miRs used.

## Discussion

### Parkinson’s Disease

PD may have a genetic component. One of the factors contributing to the development of PD is the mutation of leucine-rich repeat kinase 2 (*LRRK2*). This gene is responsible for regulating the Wingless/Int (Wnt) pathway, which plays a role in the development of the nervous system, including synapse formation and the synthesis of dopaminergic neurons [58–61]. The activation of the Wnt pathway is mediated by miR-23b-3p. It was observed that PD models show a decreased level of miR-23b-3p in plasma, but an increased level in MSCs-Exo. Therefore, MSCs-Exo may exert therapeutic effects by delivering miR-23b-3p, which contributes to symptom alleviation [22].

Other relevant genes involved in PD development are *PARKIN* and *DJ-1*, which regulate inflammation and oxidative stress, and exert protective effects on dopaminergic neurons [62]. It was noted that miR-34b correlates positively with PARKIN and DJ-1, which are reduced in PD models [23]. In addition, the following have anti-inflammatory actions: miR-188-3p and miR-494-3p [27, 28], and anti-oxidative actions: miR-100-5p and miR-181a-2-3p [24, 25].

The anti-oxidative effect of miR-100-5p is associated with a reduction in Nox 4 level [24]. Reducing the abundance of this protein decreases the amount of oxidative stress products. Moreover, Nox4 contributes to hypersensitivity seen in PD models. Inhibition of Nox activation has been found to alleviate pain associated with PD [62].

Anti-inflammatory action has specific significance for potential treatment. Inflammation contributes to the induction of apoptosis in dopaminergic neurons, which is one of the main causes of PD symptoms [60]. The RhoA-ROCK pathway plays a role in the onset of neuroinflammation [63]. Inhibition of this pathway is mediated by miR-133b [30]. Furthermore, reduction of neuronal death may be triggered by anti-apoptotic miRs such as miR-181a-2-3p and miR-188-3p [25, 27]. However, it is necessary to take into account that some miRs have opposite effects. For instance, miR-23b-3p increases neuronal autophagy, while miR-155 induces apoptosis [22, 26].

Excessive production of α-syn is observed in PD models [60]. α-syn is a protein that forms neurotoxic aggregates in the brain [64]. This review demonstrates that certain types of miRs may reduce α-syn levels. These include miR-23b-3p, miR-34b, miR-181a-2-3p, miR-188-3p, and miR-149-5p [22, 23, 25, 27, 29]. Overexpression of these miRs in MSCs may represent a potential therapeutic target. Nonetheless, it was found that reduction of α-syn, when applied as an isolated intervention, has no significant effect on alleviating PD symptoms [60, 65].

TH, alongside α-syn, serves as an indicator of PD progression. This is because TH is a marker of dopaminergic neurons, and its expression reflects their number. We observed a decrease in TH levels in PD, thus reversing this effect may have therapeutic potential. It was noted that MSCs-Exo injection raises TH expression, and further enrichment of exosomes with miR-181a-2-3p boosts TH level even more [25]. Moreover, miR-100-5p, miR-188-3p, and miR-133b exhibit similar effects [24, 27, 30].

The most important aspect of PD therapy is improving motor function. It has been observed that the injection of MSCs or MSCs-Exo enriched with specific miRs may positively influence behavioral test outcomes. The collected studies employed various behavioral tests, including open field test, rotarod test, hang test, pole test, rod-climbing test and Morris Water maze test. It has been confirmed that miR-100-5p, miR-181a-2-3p, miR-188-3p, miR-149-5p, and miR-133b derived from MSCs improve behavioral test results [24, 25, 27, 29, 30]. It follows that miRs may be an important component of medications used in PD treatment.

Beyond molecular and behavioral endpoints, emerging computational studies demonstrate that pathological beta oscillations can be bidirectionally modulated within basal ganglia circuits [66]; incorporating such electrophysiological and network-level measures into preclinical miRNA studies may provide more sensitive and mechanistically informative outcome parameters than behavioral testing alone.

In the same direction, unified computational frameworks have demonstrated multimodal bifurcation control of parkinsonian beta oscillations through pedunculopontine nucleus pathways, identifying dynamic therapeutic targets beyond conventional dopaminergic strategies [67]. Integrating such network-level endpoints with MSCs-miRNA therapy could clarify whether molecular neuroprotection translates into restored circuit stability.

### Alzheimer’S Disease

One of the causes of AD is the deposition of β-amyloid in the brain. β-amyloid induces oxidative stress, which leads to the death of surrounding neurons [68]. This cytotoxicity may be modulated by certain miRs. It was found that miR-29c-3p delivered by MSCs reduces β-amyloid levels [33]. In turn, miR-29 targets *BIM*. It was noted that suppression of BIM expression reduces apoptosis caused by β-amyloid [37, 69]. Furthermore, miR-223 directly contributes to a decrease in the apoptosis rate [35].

Neuronal apoptosis is further induced by the inflammatory process developing during the course of AD [70]. MSCs through certain miRs including miR-146a, miR-22, miR-223, and miR-223-3p may reduce inflammation, thereby leading to the alleviation of AD symptoms [31, 34, 35, 38].

Pyroptosis is another type of cell death that is involved in the pathomechanism of AD. Although it is a physiological process, excessive activation may contribute to the development of inflammation. It was also shown that pyroptosis participates in AD progression [69]. This process is suppressed by miR-22 and miR-223-3p. MiR-223-3p reduces pyroptosis and neuroinflammation by inhibiting *NLRP3*, thereby, potentially reducing the progression of AD [34, 38].

Consistent with this NLRP3-centered therapeutic logic, pharmacological promotion of SKP1-Cullin1-F-box E3 ligase-mediated NLRP3 ubiquitination has been shown to ameliorate macrophage pyroptosis and inflammatory pathology [71], suggesting that miRNA-mediated suppression of NLRP3 and direct targeting of NLRP3 protein turnover may act on the same upstream inflammatory axis.

The TRAF6/NF-κB pathway is one of the mechanisms involved in neuronal degradation. Inhibiting IL-17 has been shown to downregulate this pathway. This leads to improved cognitive functions in AD models [72]. It was observed that miR-146a acts similarly by decreasing the expression of TRAF6 and NF-κB in astrocytes [31].

Considering the series of neurodegenerative processes, including the progression of AD, it is important to stimulate neurodevelopmental mechanisms that may help mitigate the resulting damage. To achieve this, MSCs may be useful through the delivery of specific miRs. It has been demonstrated that miR-29c-3p activates the Wnt pathway, which supports neurodevelopment. This is because miR-29c-3p targets BACE1, which functions as aninhibitorof the Wnt signaling pathway [33]. Another miR that inactivates *BACE1* and stimulates the Wnt is miR-29 [37].

*PTEN* plays a significant role in the pathomechanism of AD. It has been shown that PTEN suppresses the PI3K/Akt pathway, which is critically involved in maintaining cellular survival and promoting cell growth [73]. Thus, inhibition of *PTEN*, for instance, through the action of miR-223, may exert neuroprotective effects [35].

Some miRs have detrimental effects. In such cases, it may be beneficial to inhibit their actions. For instance, inhibition of miR-937 increase Brn-4 and BDNF levels—factors that promote neurodevelopment and decrease the level of β-amyloid [32]. It may also be advantageous to suppress miR-211-5p expression. MiR-211-5p acts in a pro-inflammatory manner, increases the rate of apoptosis and cell migration, which may exacerbate AD symptoms. In addition, miR-211-5p downregulates NEP enzyme activity, impairing β-amyloid removal [36, 74].

Delivery of miRs via MSCs may improve motor functions. It has been observed that miR-22, miR-29, and miR-214-3p improve behavioral tests outcomes [34, 37, 39]. This suggests that miRs may become valuable tools in AD therapy, and MSCs are excellent carriers for these molecules.

### Stroke

Even brief hypoxia of brain tissue can lead to neuronal necrosis. Neuronal dysfunction results in clinical manifestations of nervous system impairment. Moreover, during stroke, inflammation and oxidative stress develop, further exacerbate cell death [75]. A higher degree of cellular dysfunction correlates with greater symptom severity. Therefore, it is crucial to implement therapeutic strategies aimed at mitigating both inflammation and oxidative stress.

It has been observed that miR-146a-5p, miR-25-3p and miR-150-5p, positively influence cell viability by reducing apoptosis, oxidative stress, and inflammation [44, 47, 48, 52]. At a mechanistic level, SIRT1 has emerged as a central nodal regulator of multiple forms of regulated cell death in ischemic stroke, including apoptosis, pyroptosis, and ferroptosis [76]; several miRNAs delivered by MSCs-derived exosomes may therefore exert their neuroprotective effects, at least in part, through convergence on SIRT1-dependent survival pathways. On the other hand, miR-206, miR-21-3p, and miR-148b-3p exhibit opposing effect, and they may exacerbate symptoms of stroke [43, 46, 51].

Stroke is a neurological disorder caused by vascular lesions. Therefore, to enable effective regeneration, proper revascularization of the damaged tissue must be restored. Pro-angiogenic miRs, such as miR-21-5p and miR-486, may support vascular regeneration [45, 54].

This dual neurogenic–angiogenic strategy is further supported by biomaterial-based approaches: neural stem cells engineered with layer-by-layer assembled VEGF reservoirs have been shown to promote both neurogenesis and angiogenesis after ischemic stroke in mice [77], suggesting that combining MSC-derived pro-angiogenic miRNAs with growth-factor-reservoir biomaterials may represent a rational next-generation therapeutic design.

The negative consequences of stroke may also affect motor neurons, leading to limb paresis [78]. Due to the long-term nature of nerve tissue regeneration, it is important to implement therapies that support this process [37]. The use of specific miRs may accelerate the reconstruction of damaged neuronal connections. For instance, miR-17–92, and miR-133b have been shown to promote axonal density and plasticity. Furthermore, miR-133b and miR-17–92 enhance synaptophysin expression, a marker of synaptic plasticity. All of these factors contribute to the improvement of motor function [41, 42, 49, 50].

The key role of miRs is to inhibit gene expression and indirectly regulate protein levels. It was demonstrated that miR-17–92 and miR-486 decrease PTEN levels, thereby exerting neuroprotecting effects [49, 55]. In addition, MiR-17–92 enhances GAP43 expression [41]. It was shown that this protein is associated with nerve growth factor (NGF) and BDNF [73, 79]. Moreover, the decrease in BNIP3 expression induced by miR-25-3p reduces neuronal autophagy [47, 80]. Additionally, miR-133b suppresses CTGF [50], which is linked to brain injury, and its inhibition may exert a neuroprotective effect [81].

### Paracrine Effects of Mscs: The Secretome and Exosome

The present review summarizes studies investigating the effects of MSCs-derived miRs in experimental models of PD, AD, and stroke. Across the included studies, miRs were delivered using two main approaches. In the first, target models were treated with MSCs, either by direct administration or co-culture, allowing the effects of the entire MSCs secretome to be evaluated [24, 26, 29, 31, 32]. In the second, treatment involved MSCs-Exo, administered either by injection or co-culture, thereby assessing the effects of only a specific fraction of the MSCs secretome [22, 23, 25, 27, 28].

Based on the currently available evidence, it is not possible to determine whether one delivery strategy is superior to the other. Nevertheless, beneficial effects were consistently observed following both MSCs- and MSCs-Exo-based approaches, suggesting that each may serve as an effective vehicle for the delivery of therapeutic miRs.

It should be noted, however, that the MSCs secretome comprises a wide range of bioactive molecules, including growth factors, cytokines, chemokines, and hormones, all of which may have contributed to the observed therapeutic effects independently of miRs [82]. In contrast, MSCs-Exo are nanosized extracellular vesicles that participate in intercellular communication through the transfer of proteins, lipids, and genetic material, including miRs. They can serve as natural carriers of therapeutic agents, and their miRs cargo exhibits greater stability compared to free cellular miRs. Moreover, exosomes are capable of crossing the blood–brain barrier and demonstrate low immunogenicity and biodegradability, making them an attractive therapeutic tool [83].

Previous studies have demonstrated that the MSCs secretome depleted of MSCs-Exo exhibits stronger immunomodulatory properties than isolated MSCs-Exo alone [84]. In addition, the MSCs secretome contains numerous neurotrophic factors, including brain-derived neurotrophic factor (BDNF) and nerve growth factor (NGF), which may further contribute to its therapeutic effects [82]. Conversely, Doeppner et al. reported no significant differences between treatment with MSCs and MSCs-Exo in a mouse model of stroke [85].

From the perspective of the present review, however, and specifically with regard to miRs-mediated effects, the majority of therapeutic miRs are contained within exosomes. Consequently, exosomes are likely to represent the principal mediators responsible for the transfer of miRs to recipient cells and the subsequent modulation of target gene expression [82, 86].

Furthermore, MSCs-Exo offer several practical advantages over MSCs with respect to storage and clinical application. Current evidence suggests that MSCs-Exo can be stored at − 80 °C [87], although standardized storage protocols have not yet been established. Unlike MSCs, exosomes do not require cryoprotective agents, which may exhibit cytotoxic effects during cryopreservation. In contrast, MSCs would lose much of their biological activity under comparable storage conditions.

In addition to these logistical advantages, Vizoso et al. highlighted several potential safety benefits of MSCs-Exo over MSCs, including a lower risk of eliciting immune responses, tumorigenesis, embolic events, and pathogen transmission [88]. These characteristics make MSCs-Exo an attractive cell-free therapeutic platform for future clinical applications.

One of the major challenges associated with MSCs-based therapy is the limited availability and persistence of viable cells. Following transplantation, MSCs exhibit limited survival and are gradually cleared from the host, necessitating repeated administrations to maintain therapeutic efficacy. In addition, current isolation techniques remain insufficient to consistently yield the large numbers of MSCs required for clinical applications. Although in vitro expansion can increase cell numbers, prolonged cell culture introduces additional challenges, including the risk of contamination with culture reagents and alterations in MSCs phenotype and biological properties resulting from prolonged passaging and spontaneous differentiation.

In contrast, MSCs-Exo offer greater scalability, as they can be produced in large quantities using optimized cell culture systems [88]. This manufacturing potential, together with their favorable storage characteristics and safety profile, further supports the development of MSCs-Exo as a promising cell-free therapeutic alternative.

It should be emphasized that the successful clinical translation of MSCs-Exo requires the standardization of methods for exosome isolation, characterization, purification, and storage. Such standardization is essential to ensure the reproducibility, comparability, and reliability of preclinical and clinical findings.

Current recommendations for the isolation and characterization of extracellular vesicles are provided in the 2018 Minimal Information for Studies of Extracellular Vesicles (MISEV2018) guidelines [89]. However, Eleuteri and Fierabracci have emphasized that further research is needed to optimize and validate existing technologies before their widespread clinical implementation [90].

This issue is particularly relevant in the context of the present review, as the included studies investigated MSCs-Exo isolated from a variety of tissue sources using different isolation protocols. Therefore, the establishment of standardized procedures for exosome isolation and characterization should be considered a prerequisite for improving the reproducibility of experimental findings and facilitating the translation of MSCs-Exo therapies into clinical practice.

### Application of Cell-free Therapeutic Approaches

The collected studies employed various routes of administration of MSCs or MSCs-Exo. In animal models, the most commonly used delivery routes were intravenous administration of MSCs/MSCs-Exo [34, 39, 41, 43–45] or intracerebral administration [31–33, 38]. Both approaches have been shown to be effective, however, each is associated with distinct limitations. Intracerebral administration enables bypassing the blood–brain barrier and ensures rapid and direct delivery of the therapeutic agent to the site of injury, nevertheless, it is a highly invasive procedure associated with significant clinical risk.

In contrast, intravenous administration represents a minimally invasive approach, allowing for repeated applications, which is particularly relevant in the context of MSCs that are gradually degraded in the recipient’s organism and may require repeated supplementation. However, this route requires crossing the blood–brain barrier, which limits central nervous system bioavailability, prolongs the time required to reach the site of injury, and is associated with the risk of partial loss of cells or vesicles during systemic distribution.

One of the major challenges in the treatment of neurodegenerative and cerebrovascular diseases is the presence of the blood–brain barrier, which significantly restricts the passage of many therapeutic agents into neural tissue. Consequently, alternative routes of administration are being explored. One such approach is direct intracerebral delivery, however, this method remains highly invasive and is associated with a considerable risk of complications.

An alternative approach includes intravenous or intranasal administration (via the nasal epithelium). Intranasal delivery enables partial bypass of the blood–brain barrier through transport along the olfactory and trigeminal nerves, however, it requires specific formulation properties such as appropriate solubility, pH, and osmolarity. Additionally, there is a risk of drug deposition in the respiratory tract, which may reduce the efficiency of delivery to the central nervous system [91].

Intravenous administration remains an effective option, particularly for molecules capable of crossing the blood–brain barrier, such as microvesicles, nanocarriers, or exosomes, which are widely represented in the studies included in this review. In the present context, particular attention has been given to microRNAs (miRs) transported within MSCs-Exo, which exhibit neuroprotective, anti-inflammatory, and antioxidant properties and may also support the integrity of the blood–brain barrier, which is of key importance in the treatment AD, PD, and stroke [92].

The use of MSCs-Exo in the treatment of neurological disorders appears particularly promising. However, most studies on PD and AD are currently limited to animal models and cell lines. A more advanced stage of research is observed in stroke, where clinical studies are already underway. Bang et al. demonstrated that administration of MSCs into the bloodstream of stroke patients is associated with a correlation between circulating exosome levels and improvement in motor function [93]. Furthermore, a clinical trial evaluating the intranasal administration of MSCs-Exo in stroke patients (ID: NCT05158101) has been registered and, at the time of writing this manuscript (23 June 2026), remains in the participant recruitment phase (https://clinicaltrials.gov/study/NCT05158101).

In conclusion, the use of exosomes as therapeutic carriers represents a promising strategy to overcome the limited permeability of the blood–brain barrier, which remains one of the major challenges in the treatment of neurological diseases and brain injuries. Nevertheless, most available evidence is derived from preclinical studies, and the number of well-documented clinical trials remains limited.

### Study Limitations

The present systematic review provides promising evidence supporting the therapeutic potential of MSCs-derived miRs, however, several limitations should be considered when interpreting the findings. First, the overall number of studies investigating this specific therapeutic approach remains relatively limited for each of the neurological disorders included in this review. Second, substantial heterogeneity was observed among the included studies. Different miRs were evaluated, and the exosomes used originated from various MSCs sources. Furthermore, considerable variability existed in the intervention protocols, routes of administration, treatment regimens, and experimental designs, as well as in the selection of control groups. Consequently, direct comparisons between studies and the performance of a quantitative meta-analysis were not feasible.

Third, the in vivo studies generally included relatively small sample sizes, ranging from 3 to 42 animals per experimental group, which may have limited the statistical power and robustness of the reported findings. In addition, many of the included studies did not clearly report methodological measures aimed at minimizing the risk of bias, such as random allocation of animals to experimental groups or blinding of outcome assessors. The lack of these procedures may increase the likelihood of systematic bias, particularly in studies evaluating behavioral outcomes.

Finally, all studies included in this review were preclinical, relying exclusively on animal models and in vitro cell culture systems. Therefore, caution should be exercised when extrapolating these findings to clinical practice. Although the available evidence highlights the considerable therapeutic potential of MSCs-Exo miRs, well-designed clinical trials are still required to confirm their safety, efficacy, optimal dosing strategies, and long-term therapeutic benefits in patients with neurological disorders.

## Conclusion

The studies evaluated in this systematic review demonstrate that MSCs- and MSCs-Exo-based interventions hold substantial therapeutic potential for the treatment of neurodegenerative diseases and stroke. While studies employing isolated or miRNAs-loaded exosomes provide direct evidence of miRNAs-specific neuroprotective mechanisms, the benefits observed in whole-MSCs models likely reflect synergistic effects of miRNAs and other complex components of the secretome. The analysis identified several miRNAs with potential therapeutic benefits, with miR-133b, miR-146a, miR-17–92, and miR-223 being the most frequently reported.

The greatest hopes lie with miRs derived from exosomes secreted by MSCs due to their ability to cross the blood–brain barrier, opening new perspectives for the treatment of diseases for which effective therapies are currently lacking. However, before this can happen, more extensive research is needed. Currently, research is focused on animal and cell models; there is a need for broader studies with transfer to human models, as well as the creation of safe, reproducible protocols for the isolation and subsequent administration of selected miRs to patients suffering from neurodegenerative diseases. 

## Supplementary Information

Below is the link to the electronic supplementary material.

ESM 1 (DOCX 211 KB)

ESM 2 (PDF 441 KB)

ESM 3 (PNG 1.30 MB)

## Data Availability

All data generated or analyzed during this study are included in this published article. The systematic review protocol is registered and publicly available in the Open Science Framework (OSF) repository at https://osf.io/cxt9b.
