# Supplementary material for: The Therapeutic Potential of MicroRNAs Delivered By Mesenchymal Stem Cells in Parkinson’s Disease, Alzheimer’s Disease, and Stroke: A Systematic Review of Preclinical Studies
Source: Mol Neurobiol. 2026 Oct 3;63(1):944. doi: 10.1007/s12035-026-06247-3 (PMC13633960; doi:10.1007/s12035-026-06247-3)
Supplement: Supplementary file 1 [file 12035_2026_6247_MOESM2_ESM.pdf]

OHAT Risk of Bias Rating Tool for Human and Animal Studies

INTRODUCTION

This document is written to outline a tool for evaluating individual study risk of bias or internal validity – the assessment of whether the design and conduct of a study compromised the credibility of the link between exposure and outcome (Higgins and Green 2011, IOM 2011, Viswanathan *et al.* 2012). The risk-of-bias rating tool presents a parallel approach to evaluating risk of bias in human and non-human animal studies to facilitate consideration of risk of bias across elements and across evidence streams with common terms and categories.

This tool was developed based on the most recent guidance from the Agency for Healthcare Research and Quality (Viswanathan *et al.* 2012, 2013), the Cochrane risk-of-bias tool for non-randomized studies of interventions (Sterne *et al.* 2014), Cochrane Handbook (Higgins and Green 2011), CLARITY Group at McMaster University (2013), SYRCLE's risk-of-bias tool for animal studies (Hooijmans *et al.* 2014), the Navigation Guide (Johnson *et al.* 2013, Koustas *et al.* 2013, Johnson *et al.* 2014, Koustas *et al.* 2014, Woodruff and Sutton 2014), comments from the public and technical advisors on draft methods and risk-of-bias instructions (NTP 2013d, c, b, a), staff at other federal agencies, and other sources (Downs and Black 1998, Genaidy *et al.* 2007, Dwan *et al.* 2010, Shamliyan *et al.* 2010, Shamliyan *et al.* 2011, Krauth *et al.* 2013, Wells *et al.* 2014).

For each study, risk of bias is assessed at the outcome level because certain aspects of study design and conduct may increase risk of bias for some outcomes and not others within the same study.

Organization of This Document

The majority of this document is devoted to providing detailed instructions for rating risk of bias of individual studies. Potential sources of bias are assessed with a set of 10 questions or "domains" and an additional category to consider "other potential threats to internal validity." Study design determines which questions apply [e.g., questions #1, 2, 5, 6, 7, 8, 9, 10 and 11 (or "other") apply to experimental animal studies with a different set for case-control human studies]. Detailed criteria are provided under each question that are specific for each study design. The instructions outline criteria by which individual studies are assessed and define aspects of study design, conduct, and reporting that are used to assign a risk-of-bias rating for each question.

The introduction section includes clarification of risk of bias relative to indirectness and other factors that are not considered within the OHAT risk-of-bias framework. It also provides suggestions for customizing the risk-of-bias criteria for a specific research question.

Indirectness, Timing, and Other Factors Related to Risk of Bias

Risk of bias vs indirectness:

This risk-of-bias tool evaluates internal validity – the assessment of whether the design and conduct of the study compromised the credibility of the link between exposure and outcome (Higgins and Green 2011, IOM 2011, Viswanathan *et al.* 2012). There are other aspects of a study that will impact its utility for addressing the research question such as external validity – indirectness or applicability, which are addressed elsewhere in the OHAT Approach. In other words, risk of bias addresses the question "Are the results of the study credible?" Whereas indirectness addresses the question "Did the study design address the topic of the evaluation?"

It is useful to note that some study features may be relevant to risk of bias and indirectness (Viswanathan *et al.* 2012). In particular, there are several aspects of a study relating to time, that need to be considered in both risk of bias and indirectness. For example, if there are differences in the duration of follow up across study groups, this would be a source of bias considered under detection bias "Can we be confident in the outcome assessment?" That same duration of follow up is also relevant to the indirectness or applicability of a study. If the duration of follow up was not sufficient for the development of the outcome of interest (e.g., a 6-week study of cancer endpoints), then an otherwise well-designed and well-conducted study may suffer from indirectness despite having low risk of bias.

These interrelated factors regarding timing of exposure and outcome can be considered at multiple places during an evaluation.

Time-related factors are considered at 4 points in the OHAT Approach:

- Eligibility criteria for selecting studies in Step 2 can exclude studies *a priori* where the timing of the exposure or outcome assessment are clearly inappropriate for consideration in an evaluation (e.g., chronic endpoints assessed in an acute exposure study).
- A risk-of-bias question under detection bias "Can we be confident in the exposure assessment?" considers if the exposure was assessed at a consistent time point across study groups.
- A risk-of-bias question under detection bias "Can we be confident in the outcome assessment?" considers if the outcome was assessed at a consistent time point across study groups.
- And under indirectness and applicability in Step 5 considers if the timing of exposure and outcome is acceptable for the evaluation.

The following questions are addressed in rating confidence in the body of evidence (Step 5 of the OHAT Approach), not in the risk-of-bias assessment:

- Did exposure assessment represent exposures that occurred prior to the development of the outcome? This is considered as a key feature of study design for the initial confidence rating.
- Was the exposure in the appropriate biological window to affect the outcome? This is considered under indirectness.
- Was the outcome assessed at an adequate amount of time after the exposure for the development of the outcome? This is considered under indirectness.
- Does the timing of exposure or outcome assessment impact the consistency of results? If the appropriate biological window is unclear for an outcome of interest, differences in timing of exposure or outcome assessment could be used to stratify results when considering unexplained inconsistency.

Customizing Risk-of-bias Criteria During Protocol Development

The risk-of-bias criteria and rating instructions provided in this document can be applied to many research questions, but in all cases they should be tailored to the specific research question for a given systematic review. While the criteria for most of the risk-of-bias questions will be largely similar across different reviews, the criteria for three questions should be explicitly customized for each evaluation: 1) consideration of potential confounders, 2) confidence in the exposure characterization, and 3) confidence in the outcome assessment.

Systematic review authorities recommended that subject-matter experts with knowledge of the literature participate in drafting a list of potential confounders when a review protocol is developed (Viswanathan *et al.* 2013, Sterne *et al.* 2014). Expertise and knowledge of both the exposure and outcomes of interest is required for identifying potential confounders. We recommended that experts with knowledge of the literature (including both exposure and outcome) participate in drafting the risk-of-bias criteria for potential confounders, exposure characterization, and outcome assessment when a review protocol is developed. It may be helpful to draft an analytic framework to show potential confounders that could affect the relationship between exposure and outcomes of interest. Even with early expert consultation, questions may arise when the actual studies are assessed. Additional consultation and modifications to the risk-of-bias criteria for confounders, exposure, and outcomes may be necessary. When changes are made, they should be documented along with the date on which modifications were made and the logic or justification for the changes.

Direction of Bias

Empirical evidence about the direction of bias is discussed for each of the risk-of-bias questions. Users of this document are encouraged to judge the direction of bias when possible. For some questions, the evidence will be easier to evaluate as toward or away from the null. For example, non-differential unintended co-exposure to high background phytoestrogen content in the diet will bias experimental studies of low-dose estrogenic effects toward the null. However, if there is no clear rationale for judging the likely direction of bias, review authors should simply outline the evidence and not attempt to guess the direction of evidence (Sterne *et al.* 2014).

General Instruction Format

How this tool is structured:	Study Type Abbreviations:
• 11 Risk-of-bias questions or domains<br>• Each question is applicable to 1 to 6 study design types<br>• Questions are rated by selecting among 4 possible answers (see below)<br>• Questions are grouped under 6 types of bias (selection, confounding, performance, attrition/exclusion, detection, and selective reporting)<br>• In practice, we will use web-based forms and reviewers will only see questions and instructions that are relevant to the study under review (i.e., text related to human studies will not appear during the evaluation of an animal study)	**EA:** Experimental Animal<br>**HCT:** Human Controlled Trial[1]<br>**Co:** Cohort<br>**CaCo:** Case-Control<br>**CrSe:** Cross-sectional<br>**CaS:** Case Series/Case report

[1] Human controlled trial study design used here refers to studies in humans with a controlled exposure including randomized controlled trials and non-randomized experimental studies

Question Format:

- Background
  - Definition of the general category of bias
  - Clarifying text to explain what study aspects are relevant
  - Available empirical information about the direction and magnitude of the bias
  - Information about other internal validity assessment tools that consider this element
- Specific risk-of-bias rating instructions customized to each study type
  - Detailed criteria are outlined that define aspects of the study design, conduct, and reporting required to reach each risk-of-bias rating
  - The criteria are focused on distinguishing among the 4 risk-of-bias answers or ratings (e.g., outlining factors that separate "definitely low" from "probably low" risk of bias

Answer Format:

++ ***Definitely Low*** *risk of bias*:

There is direct evidence of low risk-of-bias practices
(May include specific examples of relevant low risk-of-bias practices)

\+ ***Probably Low*** *risk of bias*:

There is indirect evidence of low risk-of-bias practices **OR** it is deemed that deviations from low risk-of-bias practices for these criteria during the study would not appreciably bias results, including consideration of direction and magnitude of bias.

– NR ***Probably High*** *risk of bias*:

There is indirect evidence of high risk-of-bias practices **OR** there is insufficient information (e.g., not reported or "NR") provided about relevant risk-of-bias practices

–– ***Definitely High*** *risk of bias*:

There is direct evidence of high risk-of-bias practices
(May include specific examples of relevant high risk-of-bias practices)

The system for answering each risk-of-bias question requires reviewers to choose between low and high risk-of-bias options. This 4-point scale is based on the approach taken by the Clarity Group at McMaster University without an answer for mixed or unclear evidence (2013). A conservative approach is taken wherein insufficient information to clearly judge the risk of bias for an individual question results in an answer rating of "Probably High" risk of bias. To clearly identify answers that were reached due to insufficient information, there are two separate symbols for "Probably High" risk of bias: 1) "-" for indirect evidence of high risk-of-bias practices, and 2) "NR" or not reported when there is insufficient information. The general answer format was adapted from (Koustas *et al.* 2013).

RISK OF BIAS RATING INSTRUCTIONS

Selection Bias

Selection bias refers to systematic differences between baseline characteristics of the groups that are compared (Higgins and Green 2011).

1. Was administered dose or exposure level adequately randomized?

Randomization of exposure or sequence generation (along with allocation concealment in question #2) helps to assure that treatment is not given selectively based on potential differences in human subjects or non-human experimental animals (e.g., randomization by animal body weight avoids potential selection bias introduced by assigning all of the smallest animals to the high-dose exposure group). Randomization requires that each human subject or animal had an equal chance of being assigned to any study group including controls (e.g., use of random number table or computer generated randomization). This applies to a concurrent negative control group (i.e., a group for which exposure is to vehicle or media alone or un-treated) which must be included in the study to address randomization as well as any positive control group that may be part of the study. For some experimental designs, the analyses are performed relative to basal levels and therefore a human subject or animal may serve as its own control.

A lack of randomization can bias results away from the null towards larger effect sizes. This effect has been empirically assessed in both controlled human trials (reviewed in Higgins and Green 2011) and experimental animals (reviewed in Krauth *et al.* 2013).

This element is widely recommended to assess risk of bias for controlled human trials (Guyatt *et al.* 2011, Higgins and Green 2011, IOM 2011, Viswanathan *et al.* 2012) and is included in most risk-of-bias instruments for animal studies (reviewed in Krauth *et al.* 2013, Hooijmans *et al.* 2014).

We recognize that given reporting practices for experimental animal studies it is unlikely that the allocation method will be explicitly reported in most studies. Thus, in cases where randomization is reported but the method is unknown (i.e., not reported and cannot be obtained through author query), we will classify studies as “probably low risk of bias”. In cases where randomization is not reported, we will assume that randomization was not undertaken and classify such studies as “probably high risk of bias”.

Note: normalization is discussed in a separate risk-of-bias question under confounding bias: Did the study design or analysis account for important confounding or modifying variables?

Applies to: HCT, EA

Definitely Low risk of bias:

HCT: There is direct evidence that subjects were allocated to any study group including controls using a method with a random component. Acceptable methods of randomization include: referring to a random number table, using a computer random number generator, coin tossing, shuffling cards or envelopes, throwing dice, or drawing of lots (Higgins and Green 2011). Restricted randomization (e.g., blocked randomization) to ensure particular allocation ratios will be considered low risk of bias. Similarly, stratified randomization and minimization approaches

that attempt to minimize imbalance between groups on important prognostic factors (e.g., body weight) will be considered acceptable.

EA: There is direct evidence that animals were allocated to any study group including controls using a method with a random component,

AND there is direct evidence that the study used a concurrent control group as an indication that randomization covered all study groups.

Note: Acceptable methods of randomization include: referring to a random number table, using a computer random number generator, coin tossing, shuffling cards or envelopes, throwing dice, or drawing of lots (Higgins and Green 2011). Restricted randomization (e.g., blocked randomization) to ensure particular allocation ratios will be considered low risk of bias. Similarly, stratified randomization and minimization approaches that attempt to minimize imbalance between groups on important prognostic factors (e.g., body weight) will be considered acceptable. This type of approach is used by NTP, i.e., random number generator with body weight as a covariate.

Note: Investigator-selection of animals from a cage is not considered random allocation because animals may not have an equal chance of being selected, e.g., investigator selecting animals with this method may inadvertently choose healthier, easier to catch, or less aggressive animals.

Probably Low risk of bias:

HCT: There is indirect evidence that subjects were allocated to study groups using a method with a random component (i.e., authors state that allocation was random, without description of the method used),

OR it is deemed that allocation without a clearly random component during the study would not appreciably bias results. For example, approaches such as biased coin or urn randomization, replacement randomization, mixed randomization, and maximal randomization may require consultation with a statistician to determine risk-of-bias rating (Higgins and Green 2011).

EA: There is indirect evidence that animals were allocated to any study group including controls using a method with a random component (i.e., authors state that allocation was random, without description of the method used),

AND there is direct or indirect evidence that the study used a concurrent control group as an indication that randomization covered all study groups,

OR it is deemed that allocation without a clearly random component during the study would not appreciably bias results. For example, approaches such as biased coin or urn randomization, replacement randomization, mixed randomization, and maximal randomization may require consultation with a statistician to determine risk-of-bias rating (Higgins and Green 2011).

Probably High risk of bias:

HCT: There is indirect evidence that subjects were allocated to study groups using a method with a non-random component,

OR there is insufficient information provided about how subjects were allocated to study groups (record "NR" as basis for answer).

Note: Non-random allocation methods may be systematic, but have the potential to allow participants or researchers to anticipate the allocation to study groups. Such "quasi-random" methods include alternation, assignment based on date of birth, case record number, or date of presentation to study (Higgins and Green 2011).

EA: There is indirect evidence that animals were allocated to study groups using a method with a non-random component,

OR there is indirect evidence that there was a lack of a concurrent control group,

OR there is insufficient information provided about how subjects were allocated to study groups (record "NR" as basis for answer).

Note: Non-random allocation methods may be systematic, but have the potential to allow researchers to anticipate the allocation of animals to study groups (Higgins and Green 2011). Such "quasi-random" methods include investigator-selection of animals from a cage, alternation, assignment based on shipment receipt date, date of birth, or animal number.

Definitely High risk of bias:

HCT: There is direct evidence that subjects were allocated to study groups using a non-random method including judgment of the clinician, preference of the participant, the results of a laboratory test or a series of tests, or availability of the intervention (Higgins and Green 2011).

EA: There is direct evidence that animals were allocated to study groups using a non-random method including judgment of the investigator, the results of a laboratory test or a series of tests (Higgins and Green 2011),

OR there is direct evidence that there was a lack of a concurrent control group, indicating that randomization did not cover all study groups.

2. Was allocation to study groups adequately concealed?

Allocation concealment prior to assigning the exposure level or treatment group (along with randomization in question #1) helps to assure that treatment is not given selectively based on potential differences in human subjects or non-human experimental animals.

Allocation concealment requires that research personnel allocating subjects or animals to treatment groups (including the control group) could not foresee which administered dose or exposure level is going to be assigned at the start of a study. Human studies also require that allocation be concealed from human subjects prior to entering the study.

A lack of allocation concealment can bias results away from the null towards larger effect sizes. This effect has been empirically assessed in both controlled human trials [(Schulz *et al.* 1995, Schulz *et al.* 2002, Pildal *et al.* 2007); see also studies reviewed in (Higgins and Green 2011)] and in animal studies [(Macleod *et al.* 2008) ; see also studies reviewed in (Krauth *et al.* 2013)].

This element is widely recommended to assess risk of bias for controlled human trials (Guyatt *et al.* 2011, Higgins and Green 2011, IOM 2011, Viswanathan *et al.* 2012) and included in some risk-of-bias instruments for animal studies (reviewed in Krauth *et al.* 2013).

Note: there are separate risk-of-bias issues affected by concealment or blinding that are important for selection, performance and detection bias: 1) a question under performance bias addresses blinding of research personnel and human subjects to study groups during the study; and 2) a question under detection bias addresses blinding during outcome assessment.

Applies to: HCT, EA

Definitely Low risk of bias:

HCT: There is direct evidence that at the time of recruitment the research personnel and subjects did not know what study group subjects were allocated to, and it is unlikely that they could have broken the blinding of allocation until after recruitment was complete and irrevocable. Acceptable methods used to ensure allocation concealment include central allocation (including telephone, web-based and pharmacy-controlled randomization); sequentially numbered drug containers of identical appearance; sequentially numbered, opaque, sealed envelopes; or equivalent methods.

EA: There is direct evidence that at the time of assigning study groups the research personnel did not know what group animals were allocated to, and it is unlikely that they could have broken the blinding of allocation until after assignment was complete and irrevocable. Acceptable methods used to ensure allocation concealment include sequentially numbered treatment containers of identical appearance or equivalent methods.

Probably Low risk of bias:

HCT: There is indirect evidence that the research personnel and subjects did not know what study group subjects were allocated to and it is unlikely that they could have broken the blinding of allocation until after recruitment was complete and irrevocable,

OR it is deemed that lack of adequate allocation concealment would not appreciably bias results.

EA: There is indirect evidence that at the time of assigning study groups the research personnel did not know what group animals were allocated to and it is unlikely that they could have broken the blinding of allocation until after assignment was complete and irrevocable,

OR it is deemed that lack of adequate allocation concealment would not appreciably bias results.

Probably High risk of bias:

HCT: There is indirect evidence that at the time of recruitment it was possible for the research personnel and subjects to know what study group subjects were allocated to, or it is likely that they could have broken the blinding of allocation before recruitment was complete and irrevocable,

OR there is insufficient information provided about allocation to study groups (record "NR" as basis for answer).

Note: Inadequate methods include using an open random allocation schedule (e.g., a list of random numbers); assignment envelopes used without appropriate safeguards (e.g., if envelopes were unsealed or non-opaque or not sequentially numbered); alternation or rotation; date of birth; case record number; or any other explicitly unconcealed procedure. For example, if the use of assignment envelopes is described, but it remains unclear whether envelopes were sequentially numbered, opaque and sealed.

EA: There is indirect evidence that at the time of assigning study groups it was possible for the research personnel to know what group animals were allocated to, or it is likely that they could have broken the blinding of allocation before assignment was complete and irrevocable,

OR there is insufficient information provided about allocation to study groups (record “NR” as basis for answer).

Definitely High risk of bias:

HCT: There is direct evidence that at the time of recruitment it was possible for the research personnel and subjects to know what study group subjects were allocated to, or it is likely that they could have broken the blinding of allocation before recruitment was complete and irrevocable.

EA: There is direct evidence that at the time of assigning study groups it was possible for the research personnel to know what group animals were allocated to, or it is likely that they could have broken the blinding of allocation before assignment was complete and irrevocable.

3. Did selection of study participants result in appropriate comparison groups?

Comparison group appropriateness refers to having similar baseline characteristics of factors related to the outcome measures of interest between groups aside from the exposures (and outcomes for case-control studies).

Assessment of appropriate selection of comparison groups is a widely used element of tools to assess study quality for observational human studies (Downs and Black 1998, Shamliyan *et al.* 2010, Viswanathan *et al.* 2012, CLARITY Group at McMaster University 2013, Sterne *et al.* 2014, Wells *et al.* 2014). This question addresses whether exposed and unexposed subjects were recruited from the same populations in cohort or cross-sectional studies and consideration of appropriate selection of cases and controls in case-control studies.

The direction of the bias (towards or away from the null) will differ based on the nature of differences between comparison groups and may be difficult to predict.

For example, in occupational cohorts, it is common for workers to have lower rates of disease and mortality than the general population – the healthy worker effect – because the severely ill and chronically disabled are commonly excluded from employment (Gerstman 2013). Therefore, comparing workers to an inherently less healthy group (general population or workers with less physically demanding work) can bias the estimate of disease risk towards the null (Rothman *et al.* 2012). Conversely, if cases of disease identified from a screening program were compared to controls from the general population, the effect estimate could be overestimated as those being screened may inherently have a higher risk (e.g., family history) so the better comparison group would be subjects screened as not having disease (Szklo and Nieto 2007).

For controlled exposure studies (i.e., experimental human or animal studies), the potential for imbalance of baseline characteristics is controlled for through randomization and allocation concealment. Imbalance can arise from chance alone, but baseline characteristics should be similar for truly randomized human controlled trials (Higgins and Green 2011) or other experimental studies. The majority of study quality tools for experimental animals do not have a separate question on baseline characteristics (Krauth *et al.* 2013, Koustas *et al.* 2014); although the SYRCLE tool asks whether groups were “similar at baseline or were they adjusted for confounders in the analysis” (Hooijmans *et al.* 2014). The Cochrane risk-of-bias tool for randomized controlled trials does not include a routine question on baseline characteristics, and instead suggests that reviewers consider “inexplicable baseline imbalance” under other potential threats to internal validity (Higgins *et al.* 2011). This tool takes the same approach for all experimental studies and addresses baseline imbalance for these studies only where it is strongly

suspected with a question at the end of the risk-of-bias-tool under other potential threats to internal validity.

Applies to: Co, CaCo, CrSe [HCT and EA see other potential threats to internal validity]

Definitely Low risk of bias:

Co, CrSe: There is direct evidence that subjects (both exposed and non-exposed) were similar (e.g., recruited from the same eligible population, recruited with the same method of ascertainment using the same inclusion and exclusion criteria, and were of similar age and health status), recruited within the same time frame, and had the similar participation/response rates.

CaCo: There is direct evidence that cases and controls were similar (e.g., recruited from the same eligible population including being of similar age, gender, ethnicity, and eligibility criteria other than outcome of interest as appropriate), recruited within the same time frame, and controls are described as having no history of the outcome.

Note: A study will be considered low risk of bias if baseline characteristics of groups differed but these differences were considered as potential confounding or stratification variables (see question #4).

Probably Low risk of bias:

Co, CrSe: There is indirect evidence that subjects (both exposed and non-exposed) were similar (e.g., recruited from the same eligible population, recruited with the same method of ascertainment using the same inclusion and exclusion criteria, and were of similar age and health status), recruited within the same time frame, and had the similar participation/response rates,
OR differences between groups would not appreciably bias results.

CaCo: There is indirect evidence that cases and controls were similar (e.g., recruited from the same eligible population, recruited with the same method of ascertainment using the same inclusion and exclusion criteria, and were of similar age), recruited within the same time frame, and controls are described as having no history of the outcome,
OR differences between cases and controls would not appreciably bias results.

Probably High risk of bias:

Co, CrSe: There is indirect evidence that subjects (both exposed and non-exposed) were not similar, recruited within very different time frames, or had the very different participation/response rates,
OR there is insufficient information provided about the comparison group including a different rate of non-response without an explanation (record "NR" as basis for answer).

CaCo: There is direct evidence that controls were drawn from a very dissimilar population than cases or recruited within very different time frames,
OR there is insufficient information provided about the appropriateness of controls including rate of response reported for cases only (record "NR" as basis for answer).

Definitely High risk of bias:

Co, CrSe: There is direct evidence that subjects (both exposed and non-exposed) were not similar, recruited within very different time frames, or had the very different participation/response rates.

CaCo: There is direct evidence that controls were drawn from a very dissimilar population than cases or recruited within very different time frames.

Confounding Bias

Bias relating to confounding and co-exposures is addressed under selection bias and performance in study quality tools such as Cochrane, AHRQ, and SYRCLE (Higgins and Green 2011, Higgins *et al.* 2011, Viswanathan *et al.* 2012, Hooijmans *et al.* 2014). The grouping of these related factors under "confounding bias" does not change the questions or the evaluation of bias, but rather is done for clarity in communicating bias related to confounding, modifying variables, and other exposures that are anticipated to bias results.

4. Did the study design or analysis account for important confounding and modifying variables?

Interpretation of study findings may be distorted by failure to consider the extent to which systematic differences in baseline characteristics risk factors, prognostic variables[2], or co-occurring exposures among comparison groups may reduce or increase the observed effect (IOM 2011). Confounding variables or confounders include any factor that is: 1) associated with the exposure, 2) an independent risk factor for a given outcome, and 3) unequally distributed between study groups (Gerstman 2013). The potential confounder cannot be an intermediate effect on the causal pathway between exposure and the outcome (Gerstman 2013, Sterne *et al.* 2014). Appropriate methods to account for these differences would include multivariable analysis, stratification, matching of cases and controls, or other approaches.

Adjusting or controlling for confounding is dependent on valid, reliable, and sensitive methods for assessing the confounding or modifying variables applied consistently across study groups. The requirement for assessing the confounding variables with valid and reliable measures is directly linked to the relative importance of the confounding variable considered under selection bias (i.e., if a confounder needed to be accounted for in design or analyses, then measurement of that variable had to be reliable).

This element is included in this current risk-of-bias tool because it is widely recommended in tools used to assess the quality of observational human studies (Downs and Black 1998, Shamliyan *et al.* 2010, Viswanathan *et al.* 2012, CLARITY Group at McMaster University 2013, Viswanathan *et al.* 2013, Sterne *et al.* 2014). The direction of the bias (towards or away from the null) will differ based on the nature of differences between comparison groups. Generally, confounding results in effect sizes that are

[2] "Risk" factors are those which as associated with causing a condition (like smoking for lung cancer or being born premature for chronic lung disease). 'Prognostic' factors are those which, in people who have the condition, influence the outcome (like resectability of tumor for lung cancer or duration of intubation for CLD). Risk factors are determined by looking at things that influence new cases ('incident' ones), while prognostic factors can only be determined by following up people who already have the disease (http://blogs.bmj.com/adc-archimedes/2009/03/09/risk-vs-prognostic-factors/) .

overestimated. However, confounding factors can lead to an underestimation of the effect of a treatment or exposure, particularly in observational studies. In other words, if the confounding variables were not present, the measured effect would have been even larger (IOM 2011).

Unintended co-exposures may represent a confounding factor if associated with exposure and the outcome of interest, or a modifying factor if they are independent of exposure, but associated with outcome. When an unintended exposure is an effect modifier, its level will alter the magnitude of the effect of the primary outcome. The direction of the bias (towards or away from the null) will differ based on the nature of unintended exposure and whether or not it is associated with the primary exposure. For example, an exposed group in a human study living at a Superfund site may also be exposed to high levels of other environmental contaminants; if these co-exposures are not accounted for in the analyses, they may bias results away from the null (towards larger effects sizes). Alternately, a co-exposure that is non-differentially distributed among both the exposed and control groups will usually bias the results toward the null by lowering precision and therefore reducing the ability to distinguish potential effects between groups based on the primary exposure.

It is understood in environmental health that people are exposed to complex mixtures of environmental contaminants and other types of exposures that make it difficult to establish chemical-specific associations. Thus, in <u>most</u> cases we will not penalize studies if other exposures or potential exposures are not adjusted or controlled for in the analyses of a target exposure. For some projects, exceptions may include studies where levels of other chemicals aside from the chemical of interest are likely to be high, such as in occupational cohorts or contaminated regions (e.g., Superfund sites). For some health outcomes, consideration of additional therapies, including medications, may also be appropriate.

By definition, confounders are specific for the outcome and the exposure. Therefore, the list of potential confounders has to be developed specifically for each evaluation and will require subject-matter expertise on both the outcome and exposure of interest. Systematic review authorities recommended that subject-matter experts with some knowledge of the literature participate in drafting a list of potential confounders when a review protocol is developed (Viswanathan *et al.* 2013, Sterne *et al.* 2014). It may be helpful to draft an analytic framework that shows potential confounders that could affect the relationship between exposure and outcomes of interest. Even when a list of potential confounders is developed when drafting the protocol, it is likely that new confounders will be identified when actually assessing the risk of bias of studies.

Although confounding is a much greater concern for observational studies, experimental studies are not entirely free of these issues. Controlled exposure studies (i.e., experimental human or animal studies) can address confounding and selection bias through study design features such as randomization and allocation concealment. Confounding by chance (i.e., confounding that is unknown, unmeasured, or poorly measured) is expected to be equally distributed between groups under true randomization; however, experimental studies may not always successfully randomize potential confounders (Viswanathan *et al.* 2013). Recognizing this, the SYRCLE risk-of-bias tool for experimental animal studies asks whether groups were “similar at baseline or were they adjusted for confounders in the analysis” (Hooijmans *et al.* 2014). The 2012 risk-of-bias guidance from AHRQ recommends consideration of confounding for randomized clinical trials largely because studies may fail to randomize confounders. However, the Cochrane risk-of-bias tool for randomized controlled trials does not include a question for confounding, nor do the majority of study quality tools for experimental animals (Krauth *et al.* 2013, Koustas *et al.* 2014).

For this tool, we have not included a separate question for confounding in experimental human or experimental animal studies because randomization and allocation concealment should address the issue of confounding. Therefore, the issue of confounding overlaps with randomization and allocation concealment, and multiple questions would address the same issue. We recognize that in some cases

confounding or effect modification may be a potential risk of bias despite procedures to address randomization. For example, confounding would be a concern if there were differential distribution of baseline characteristics such as body weight or BMI in a study of obesity, despite adequate procedures for randomization and allocation concealment. In another example, effect modification and bias toward the null would be of concern in an experimental study designed to test reproductive effects of estrogenic chemicals with non-differential co-exposures to high levels of phytoestrogens through the diet. For experimental studies where confounding is strongly suspected, randomization and allocation concealment should be addressed first. If these questions are rated "probably low" or "definitely low risk of bias," then confounding may be addressed under "other potential threats to internal validity."

Note: in the current OHAT tool, assessment of confounding requires consideration of whether or not 1) the design or analysis accounted for confounding and modifying variables, 2) the confounding variables were measured reliably and consistently, and 3) there were other exposures anticipated to bias results in reaching a single risk-of-bias rating on confounding. Previous versions of the OHAT tool used three separate questions for these factors (Did the study design or analysis account for important confounding and modifying variables?" "Were confounding variables assessed consistently across groups using valid and reliable measures" and "Did researchers adjust or control for other exposures that are anticipated to bias results?" The current tool considers these factors together because they are interrelated and recent guidance has taken a similar approach (e.g., Sterne et al. 2014).

Previous versions of the OHAT risk-of-bias tool applied the question on confounding to experimental study designs. As described above, this tool does not routinely apply this question to experimental studies because the issue of confounding overlaps with randomization and allocation concealment, and multiple questions would address the same issue. However, for review questions or individual experimental studies where confounding is strongly suspected despite adequate control for randomization and allocation concealment, confounding may be addressed under "other potential threats to internal validity."

Applies to: Co, CaCo, CrSe, CaS [HCT and EA see other potential threats to internal validity]

Definitely Low risk of bias:

Co, CrSe, CaS: There is direct evidence that appropriate adjustments or explicit considerations were made for primary covariates and confounders in the final analyses through the use of statistical models to reduce research-specific bias including standardization, matching, adjustment in multivariate model, stratification, propensity scoring, or other methods that were appropriately justified. Acceptable consideration of appropriate adjustment factors includes cases when the factor is not included in the final adjustment model because the author conducted analyses that indicated it did not need to be included,

AND there is direct evidence that primary covariates and confounders were assessed using valid and reliable measurements,

AND there is direct evidence that other exposures anticipated to bias results were not present or were appropriately measured and adjusted for. In occupational studies or studies of contaminated sites, other chemical exposures known to be associated with those settings were appropriately considered.

CaCo: There is direct evidence that appropriate adjustments were made for primary covariates and confounders in the final analyses through the use of statistical models to reduce research-specific bias including standardization, matching of cases and controls, adjustment in

multivariate model, stratification, propensity scoring, or other methods were appropriately justified,
AND there is direct evidence that primary covariates and confounders were assessed using valid and reliable measurements,
AND there is direct evidence that other exposures anticipated to bias results were not present or were appropriately measured and adjusted for.

Probably Low risk of bias:

Co, CaCo, CrSe, CaS: There is indirect evidence that appropriate adjustments were made,
OR it is deemed that not considering or only considering a partial list of covariates or confounders in the final analyses would not appreciably bias results.
AND there is evidence (direct or indirect) that primary covariates and confounders were assessed using valid and reliable measurements,
OR it is deemed that the measures used would not appreciably bias results (i.e., the authors justified the validity of the measures from previously published research),
AND there is evidence (direct or indirect) that other co-exposures anticipated to bias results were not present or were appropriately adjusted for,
OR it is deemed that co-exposures present would not appreciably bias results.
Note: As discussed above, this includes insufficient information provided on co-exposures in general population studies.

Probably High risk of bias:

Co, CrSe, CaS: There is indirect evidence that the distribution of primary covariates and known confounders differed between the groups and was not appropriately adjusted for in the final analyses,
OR there is insufficient information provided about the distribution of known confounders (record "NR" as basis for answer),
OR there is indirect evidence that primary covariates and confounders were assessed using measurements of unknown validity,
OR there is insufficient information provided about the measurement techniques used to assess primary covariates and confounders (record "NR" as basis for answer),
OR there is indirect evidence that there was an unbalanced provision of additional co-exposures across the primary study groups, which were not appropriately adjusted for,
OR there is insufficient information provided about co-exposures in occupational studies or studies of contaminated sites where high exposures to other chemical exposures would have been reasonably anticipated (record "NR" as basis for answer).

CaCo: There is indirect evidence that the distribution of primary covariates and known confounders differed between cases and controls and was not investigated further,
OR there is insufficient information provided about the distribution of known confounders in cases and controls (record "NR" as basis for answer),
OR there is indirect evidence that primary covariates and confounders were assessed using measurements of unknown validity,
OR there is insufficient information provided about the measurement techniques used (record "NR" as basis for answer),
OR there is indirect evidence that there was an unbalanced provision of additional co-exposures across cases and controls, which were not appropriately adjusted for,

OR there is insufficient information provided about co-exposures in occupational studies or studies of contaminated sites where high exposures to other chemical exposures would have been reasonably anticipated (record “NR” as basis for answer).

Definitely High risk of bias:

Co, CrSe, CaS: There is direct evidence that the distribution of primary covariates and known confounders differed between the groups, confounding was demonstrated, and was not appropriately adjusted for in the final analyses,

OR there is direct evidence that primary covariates and confounders were assessed using non valid measurements,

OR there is direct evidence that there was an unbalanced provision of additional co-exposures across the primary study groups, which were not appropriately adjusted for.

CaCo: There is direct evidence that the distribution of primary covariates and known confounders differed between cases and controls, confounding was demonstrated, but was not appropriately adjusted for in the final analyses,

OR there is direct evidence that primary covariates and confounders were assessed using non valid measurements,

OR there is direct evidence that there was an unbalanced provision of additional co-exposures across cases and controls, which were not appropriately adjusted for.

Performance Bias

Performance bias refers to systematic differences in the care provided to human participants or experimental animals by study groups. Examples include contamination of the control group with the exposure or intervention, unbalanced provision of additional interventions or co-interventions, difference in co-interventions, inadequate blinding of providers and participants in human studies (Viswanathan *et al.* 2012), and inadequate blinding of research personnel to the animal’s study group (Sena *et al.* 2007).

5. Were experimental conditions identical across study groups?

Housing conditions and husbandry practices should be identical across control and experimental groups because these variables may impact the outcome of interest (Duke *et al.* 2001, Gerdin *et al.* 2012). Identical conditions include use of the same vehicle in control and experimental animals. This risk-of-bias element is included in some tools used to assess animal studies (Krauth *et al.* 2013).

We recognize that given reporting practices it is unlikely that similarity of conditions will be explicitly reported in most animal studies. Thus, we will assume unless stated otherwise that experimental conditions (other than use of appropriate vehicle for control animals) were identical across groups which will result in most studies considered “probably low risk of bias”. Thus in this tool, the rating for this risk-of-bias element will depend largely on the consistent use vehicle across treatment groups. This risk-of-bias element is unlikely to be informative for the purposes of discriminating between studies based on housing conditions or husbandry practices. However, in the long-term, especially if reporting standards improve, collecting this information may generate data that will allow us to empirically assess evidence of bias based on housing conditions or husbandry practices or to remove those features from consideration.

Applies to: EA

Definitely Low risk of bias:

EA: There is direct evidence that same vehicle was used in control and experimental animals,
AND there is direct evidence that non-treatment-related experimental conditions were identical across study groups (i.e., the study report explicitly provides this level of detail).

Probably Low risk of bias:

EA: There is indirect evidence that the same vehicle was used in control and experimental animals,
OR it is deemed that the vehicle used would not appreciably bias results.
AND as described above, identical non-treatment-related experimental conditions are assumed if authors did not report differences in housing or husbandry.

Probably High risk of bias:

EA: There is indirect evidence that the vehicle differed between control and experimental animals,
OR authors did not report the vehicle used (record "NR" as basis for answer),
OR there is indirect evidence that non-treatment-related experimental conditions were not comparable between study groups.

Definitely High risk of bias:

EA: There is direct evidence from the study report that control animals were untreated, or treated with a different vehicle than experimental animals,
OR there is direct evidence that non-treatment-related experimental conditions were not comparable between study groups.

6. Were the research personnel and human subjects blinded to the study group during the study?

Blinding requires that research personnel do not know which administered dose or exposure level the human subject or animal is being given (i.e., study group). Human studies also require blinding of the human subjects when possible.

Human introductory text: If research personnel or human subjects are not blinded to the study groups it could affect the actual outcomes of the participants due to differential behaviors across intervention groups. During the course of a study blinding of participants and research personnel is a recommended risk-of-bias element in the most recent Cochrane guidance for assessing randomized clinical trials (Higgins and Green 2011).

No empirical evidence of bias due to failure to blind during the course of a study is currently available. However, 'blind' or 'double-blind' study descriptions usually include blinding of research personnel, human subjects, or both. Without distinguishing between the different stages of blinding during the conduct of a study, lack of blinding in randomized trials has been empirically shown to be associated with larger estimations of intervention effects (on average a 9% increase in an odds ratio) (Pildal *et al.*

2007). Schulz *et al*. (1995) analyzed 250 controlled trials and found that studies that were not double-blinded had a 17% larger estimation of treatment effect, on average. If additional investigations or co-interventions occur differentially across intervention groups, bias can also be introduced by not blinding research personnel or human subjects.

For some exposures, it is not possible to entirely blind research personnel and subjects during the course of the study (an exercise intervention or patients receiving surgery). However, adherence to a strict study protocol to minimize differential behaviors by research personnel and human subjects can reduce the risk of bias. In practice, successful blinding cannot be ensured, as it can be compromised for most interventions. In some case the treatment may have side effects possibly allowing the participant to detect which intervention they received, unless the study compares interventions with similar side effects or uses an active placebo (Boutron *et al.* 2006).

Animal introductory text: Lack of blinding of research personnel could bias the results by affecting the actual outcomes of the animals in the study. This may be due to differences in handling of animals (e.g., stress-related effects) or monitoring for health outcomes. For example, an investigator may be more likely to take measures to ensure that animals in experimental groups receive the appropriate dose volume compared to animals in the control group. Lack of blinding might also lead to bias caused by additional investigations or co-interventions regardless of the type of outcomes, if these occur differentially across intervention groups (Higgins and Green 2011).

This element is recommended to assess performance bias for controlled human trials (Higgins and Green 2011) and animal studies (reviewed in Krauth *et al.* 2013), although empirical evidence of bias due to lack of blinding of research personnel during the course of the study is not currently available. Rosenthal and Lawson (1964) reported that rats that experimenters had been told were “bright” performed better than rats labeled “dull” in Skinner box learning tests, despite the fact that they were the same rats. The study design did not allow clear separation between experimenter bias introduced during handling or training from bias at outcome assessment. As discussed under detection bias, lack of blinding at outcome assessment in animal studies is associated with larger measures of the effect (Bebarta *et al.* 2003, Sena *et al.* 2007, Vesterinen *et al.* 2010).

In animal studies, blinding of study group during the course of the study is often not possible for animal welfare considerations and the need to determine if treated animals are affected relative to controls in a treatment or dose-dependent manner (examples include clinical observations and histopathologic assessment of non-neoplastic lesions). Knowledge and tracking of higher exposed animals may also be part of animal welfare practices designed to avoid suffering associated with overtly toxic treatment doses. Under some conditions it is unlikely that blinding of research personnel during the course of a study can be fully achieved. However, animal studies are in general more tightly controlled than human studies and additional measures may be taken to reduce the risk of bias, such as the generation and use of standard operating procedures, training, and randomized husbandry or handling practices (e.g., placement in the animal room, necropsy order, etc.).

Note: there are separate risk-of-bias issues affected by concealment or blinding that are important for selection, performance and detection bias: 1) a question under selection bias addresses allocation concealment of research personnel and human subjects; and 2) a question under detection bias addresses blinding during outcome assessment.

Applies to: HCT, EA

Definitely Low risk of bias:

HCT: There is direct evidence that the subjects and research personnel were adequately blinded to study group, and it is unlikely that they could have broken the blinding during the study. Methods used to ensure blinding include central allocation; sequentially numbered drug containers of identical appearance; sequentially numbered, opaque, sealed envelopes; or equivalent methods.

EA: There is direct evidence that the research personnel were adequately blinded to study group, and it is unlikely that they could have broken the blinding during the study. Methods used to ensure blinding include central allocation; sequentially numbered treatment containers of identical appearance; sequentially numbered animal cages; or equivalent methods.

Probably Low risk of bias:

HCT: There is indirect evidence that the research personnel and subjects were adequately blinded to study group, and it is unlikely that they could have broken the blinding during the study,

OR it is deemed that lack of adequate blinding during the study would not appreciably bias results.

EA: There is indirect evidence that the research personnel were adequately blinded to study group, and it is unlikely that they could have broken the blinding during the study,

OR it is deemed that lack of adequate blinding during the study would not appreciably bias results. This would include cases where blinding was not possible but research personnel took steps to minimize potential bias, such as restricting the knowledge of study group to veterinary or supervisory personnel monitoring for overt toxicity, or randomized husbandry or handling practices (e.g., placement in the animal room, necropsy order, etc.).

Probably High risk of bias:

HCT: There is indirect evidence that it was possible for research personnel or subjects to infer the study group,

OR there is insufficient information provided about blinding to study group during the study (record "NR" as basis for answer).

Note: Inadequate methods include using an open random allocation schedule (e.g., a list of random numbers), assignment envelopes used without appropriate safeguards (e.g., if envelopes were unsealed or non-opaque or not sequentially numbered), alternation or rotation; date of birth; case record number; or any other explicitly unconcealed procedure. For example, if the use of assignment envelopes is described, but it remains unclear whether envelopes were sequentially numbered, opaque and sealed.

EA: There is indirect evidence that the research personnel were not adequately blinded to study group,

OR there is insufficient information provided about blinding to study group during the study (record "NR" as basis for answer).

Definitely High risk of bias:

HCT: There is direct evidence for lack of adequate blinding of the study group including no blinding or incomplete blinding of research personnel and subjects. For some treatments, such as behavioral interventions, allocation to study groups cannot be concealed.

EA: There is direct evidence that the research personnel were not adequately blinded to study group.

Attrition/Exclusion Bias

Attrition or exclusion bias refers to systematic differences in the loss or exclusion from analyses of participants or animals from the study and how they were accounted for in the results (Viswanathan *et al.* 2012).

7. Were outcome data complete without attrition or exclusion from analysis?

Incomplete outcome data includes loss due to attrition (nonresponse, dropout, or loss to follow-up) or exclusion from analyses. The degree of bias resulting from incomplete outcome data depends on the reasons that outcomes are missing, the amount and distribution of missing data across groups, and the potential association between outcome values and likelihood of missing data (Higgins and Green 2011). The risk of bias from incomplete outcome data can be reduced if study authors address the problem in their analyses (e.g., intention to treat analysis and imputation). Exclusion of individuals or animals from analyses should be clearly reported and outliers identified with appropriate statistical procedures.

Human introductory text: Differential or overall attrition because of nonresponse, dropping out, loss to follow-up, and exclusion of participants can introduce bias when missing outcome data are related to both exposure/treatment and outcome. Those who drop out of the study or who are lost to follow-up may be systematically different from those who remain in the study. Attrition or exclusion bias can potentially change the collective (group) characteristics of the relevant groups and their observed outcomes in ways that affect study results by confounding and spurious associations (Viswanathan *et al.* 2012). This risk-of-bias element is recommended to assess controlled human trials (Higgins and Green 2011), observational human studies (Viswanathan *et al.* 2012, Sterne *et al.* 2014) and animal studies (Krauth *et al.* 2013). However, concern over bias from incomplete outcome data is mainly theoretical and most studies that have looked at whether aspects of missing data are associated with magnitude of effect estimates have not found clear evidence of bias (reviewed in Higgins and Green 2011).

Animal introductory text: Attrition or exclusion because of illness, death, or other reasons can introduce bias when missing outcome data are related to both exposure and outcome. Attrition bias can potentially change the collective (group) characteristics of the relevant groups and their observed outcomes in ways that affect study results by confounding and spurious associations (Viswanathan *et al.* 2012). This risk-of-bias element is recommended to assess controlled human trials (Higgins and Green 2011), observational human studies (Viswanathan *et al.* 2012, Sterne *et al.* 2014) and animal studies (Krauth *et al.* 2013). However, concern over bias from incomplete outcome data is mainly theoretical and most studies that have looked at whether aspects of missing data are associated with magnitude of effect estimates have not found clear evidence of bias (reviewed in Higgins and Green 2011).

Applies to: HCT, EA, Co, CaCo, CrSe

Definitely Low risk of bias:

HCT: There is direct evidence that there was no loss of subjects during the study and outcome data were complete,

OR loss of subjects (i.e., incomplete outcome data) was adequately addressed and reasons were documented when human subjects were removed from a study or analyses. Review authors should be confident that the participants included in the analysis are exactly those who were randomized into the trial. Acceptable handling of subject attrition includes: very little missing outcome data (less than 10% in each group (Genaidy *et al.* 2007)); reasons for missing subjects unlikely to be related to outcome (for survival data, censoring unlikely to be introducing bias); missing outcome data balanced in numbers across study groups, with similar reasons for missing data across groups,

OR analyses (such as intention-to-treat analysis) in which missing data have been imputed using appropriate methods(insuring that the characteristics of subjects lost to follow up or with unavailable records are described in identical way and are not significantly different from those of the study participants).

Note: Participants randomized but subsequently found not to be eligible need not always be considered as having missing outcome data (Higgins and Green 2011).

EA: There is direct evidence that loss of animals was adequately addressed and reasons were documented when animals were removed from a study. Acceptable handling of attrition includes: very little missing outcome data; reasons for missing animals unlikely to be related to outcome (or for survival data, censoring unlikely to be introducing bias); missing outcome data balanced in numbers across study groups, with similar reasons for missing data across groups; missing outcomes is not enough to impact the effect estimate,

OR missing data have been imputed using appropriate methods (insuring that characteristics of animals are not significantly different from animals retained in the analysis).

Co: There is direct evidence that loss of subjects (i.e., incomplete outcome data) was adequately addressed and reasons were documented when human subjects were removed from a study. Acceptable handling of subject attrition includes: very little missing outcome data; reasons for missing subjects unlikely to be related to outcome (for survival data, censoring unlikely to be introducing bias); missing outcome data balanced in numbers across study groups, with similar reasons for missing data across groups,

OR missing data have been imputed using appropriate methods and characteristics of subjects lost to follow up or with unavailable records are described in identical way and are not significantly different from those of the study participants.

CaCo, CrSe: There is direct evidence that exclusion of subjects from analyses was adequately addressed, and reasons were documented when subjects were removed from the study or excluded from analyses.

Probably Low risk of bias:

HCT: There is indirect evidence that loss of subjects (i.e., incomplete outcome data) was adequately addressed and reasons were documented when human subjects were removed from a study,

OR it is deemed that the proportion lost to follow-up would not appreciably bias results (less than 20% in each group (Genaidy *et al.* 2007)). This would include reports of no statistical differences in characteristics of subjects lost to follow up or with unavailable records from those

of the study participants. Generally, the higher the ratio of participants with missing data to participants with events, the greater potential there is for bias. For studies with a long duration of follow-up, some withdrawals for such reasons are inevitable.

EA: There is indirect evidence that loss of animals was adequately addressed and reasons were documented when animals were removed from a study,

OR it is deemed that the proportion lost would not appreciably bias results. This would include reports of no statistical differences in characteristics of animals removed from the study from those remaining in the study.

Co: There is indirect evidence that loss of subjects (i.e., incomplete outcome data) was adequately addressed and reasons were documented when human subjects were removed from a study,

OR it is deemed that the proportion lost to follow-up would not appreciably bias results. This would include reports of no statistical differences in characteristics of subjects lost to follow up or with unavailable records from those of the study participants. Generally, the higher the ratio of participants with missing data to participants with events, the greater potential there is for bias. For studies with a long duration of follow-up, some withdrawals for such reasons are inevitable.

CaCo, CrSe: There is indirect evidence that exclusion of subjects from analyses was adequately addressed, and reasons were documented when subjects were removed from the study or excluded from analyses.

Probably High risk of bias:

HCT: There is indirect evidence that loss of subjects (i.e., incomplete outcome data) was unacceptably large (greater than 20% in each group (Genaidy *et al.* 2007)) and not adequately addressed,

OR there is insufficient information provided about numbers of subjects lost to follow-up (record "NR" as basis for answer).

EA: There is indirect evidence that loss of animals was unacceptably large and not adequately addressed,

OR there is insufficient information provided about loss of animals (record "NR" as basis for answer).

Co: There is indirect evidence that loss of subjects (i.e., incomplete outcome data) was unacceptably large and not adequately addressed,

OR there is insufficient information provided about numbers of subjects lost to follow-up (record "NR" as basis for answer).

CaCo, CrSe: There is indirect evidence that exclusion of subjects from analyses was not adequately addressed,

OR there is insufficient information provided about why subjects were removed from the study or excluded from analyses (record "NR" as basis for answer).

Definitely High risk of bias:

HCT, Co: There is direct evidence that loss of subjects (i.e., incomplete outcome data) was unacceptably large and not adequately addressed. Unacceptable handling of subject attrition includes: reason for missing outcome data likely to be related to true outcome, with either imbalance in numbers or reasons for missing data across study groups; or potentially inappropriate application of imputation.

EA: There is direct evidence that loss of animals was unacceptably large and not adequately addressed. Unacceptable handling of attrition or exclusion includes: reason for loss is likely to be related to true outcome, with either imbalance in numbers or reasons for loss across study groups.

CaCo, CrSe: There is direct evidence that exclusion of subjects from analyses was not adequately addressed. Unacceptable handling of subject exclusion from analyses includes: reason for exclusion likely to be related to true outcome, with either imbalance in numbers or reasons for exclusion across study groups.

Detection Bias

Detection bias refers to systematic differences between experimental and control groups with regards to how outcomes and exposures are assessed (Higgins and Green 2011) and also considers validity and reliability of methods used to assess outcomes and exposures (Viswanathan *et al.* 2012).

8. Can we be confident in the exposure characterization?

Confidence in the exposure requires valid, reliable, and sensitive methods to measure exposure applied consistently across groups. Exposure misclassification or measurement error may be independent of the outcomes (non-differential) or related to the outcome of interest (differential). Non-differential measurement error of exposures will usually bias the results toward the null by lowering precision and therefore reducing the ability to distinguish potential effects between exposure levels. Therefore, this tool considers the accuracy of the exposure characterization, including both purity and stability for controlled exposure studies, as part of the risk-of-bias rating for exposure. Differential measurement error of exposures can bias the exposure-outcome relationship and result in detection bias.

Detection bias can be minimized by using valid and reliable exposure measures applied consistently across groups (i.e., under the same method and time-frame). Studies that directly measure exposure in subjects (e.g., measurement of the chemical in blood, plasma, urine, etc.) are likely to have less measurement error and less risk of bias for exposure than studies relying on indirect measures (e.g., predictions from activity patterns and microenvironment concentrations). Exposure information obtained by self-report depends on the recall of participants and differential errors in recall can attenuate, strengthen, or even invert the true relationship (White 2003). Self-reporting of exposures for case-control studies are frequently cited as leading to differential measurement errors because cases often remember past exposures better than controls (i.e., recall bias) (e.g., see Rothman *et al.* 2012). Differential measurement error could also be introduced if the exposure data for different groups come from different sources for observational studies or are taken at different time points for experimental studies.

Acceptable methods for measuring exposure will be highly exposure dependent and therefore a specific list of acceptable, inaccurate, or potentially biased methods should be developed for each evaluation and will require subject-matter expertise. It is recommended that experts with some knowledge of the literature (including exposure and outcomes) participate in drafting the risk-of-bias criteria for exposure characterization when a review protocol is developed. Even with early expert consultation and planning, exposure questions may arise when the actual studies are assessed. Additional consultation and modifications to the exposure risk-of-bias criteria may be necessary. When changes are made, they should be documented along with the date on which modifications were made and the logic for the changes.

For controlled exposure studies (i.e., experimental human or animal studies), the use of reliable methods to measure exposure depends primarily on ensuring the purity and stability of the treatment compound. Independent verification of purity would be considered best practice because the identity and purity as listed on the bottle can be inaccurate. In NTP's experience, about 3% of chemicals purchased are the wrong chemical and the inaccuracy rate of chemical labelling rises to 10% if you include inaccurate reporting of purity (unpublished, personal communication Brad Collins, NTP chemist). It is also possible that impurities may be more toxic than the compound of interest. This occurred during an NTP study of PCB 118 where analysis revealed the presence of 0.622% of the much more potent PCB 126, resulting in the study being continued as a mixture study [(NTP 2006), see page 13]. The directions below takes a conservative approach in requiring independent verification of ≥99% purity for a single substance for "definitely low" risk of bias. However, the risk of bias associated with exposure to impurities depends on the identity of the impurities and the sensitivity of the outcome of interest which could result in potential effects of those impurities on the outcome of interest. The threshold for these values should be developed for specific research questions and reflect empirical data for the substance and outcome under consideration when possible. Therefore, for some chemicals like PCBs, ≥99% purity may not be sufficient for "definitely low" risk of bias and for others the appropriate purity value may be lower.

Exposure characterization should also include verification of the compound over the course of the test period. This is particularly important if the compound is volatile or instable. For example, daily preparation of treatment solutions may be required for unstable compounds (e.g., half-lives on the order of days). Special apparatus such as flow-through systems are needed to ensure exposure to volatile compounds. For example, Durda and Preziosi (2000) suggest the use of flow-through systems in aquatic exposures to volatile compounds (e.g., those with Henry's Law values in the range of 10^{-5} atm-m^3/mol or greater).

Human introductory text: Assessment of exposure is a widely used element of tools to assess study quality for observational human studies (Downs and Black 1998, Shamliyan *et al.* 2010, Viswanathan *et al.* 2012, CLARITY Group at McMaster University 2013, Wells *et al.* 2014). Exposure is much more difficult to measure and to accurately ensure for observational studies than for controlled exposure studies. Therefore, exposure measurement error and misclassification are more likely to contribute to risk of bias for observational studies.

The direction of the bias (towards or away from the null) will differ based on the nature of differences between comparison groups and may be difficult to predict. Non-differential misclassification of exposure will generally bias results towards the null, but differential misclassification can bias towards or away from the null, making it difficult to predict the direction of effect (Szklo and Nieto 2007). For controlled exposure studies, noncompliance with the allocated treatment could introduce differential misclassification if compliance was unequal across study groups. Adherence to a strict study protocol that includes measures to assure or assess compliance can reduce the risk of bias.

Animal introductory text: For laboratory or experimental animal studies, exposure assessment has only been included in a few (e.g., Durda and Preziosi 2000) study quality or risk-of-bias tools (reviewed in Krauth *et al.* 2013). However, as described above, this tool considers the accuracy of the exposure characterization as part of the risk-of-bias rating because non-differential exposure misclassification tends to bias the results toward the null. Wildlife or environmental-exposure animal studies are analogous to human observational studies and therefore inclusion of this element would be expected based on guidance for human studies.

Applies to: HCT, EA, Co, CaCo, CrSe, CaS

Definitely Low risk of bias:

HCT, EA: There is direct evidence that the exposure (including purity and stability of the test substance and compliance with the treatment, if applicable) was independently characterized and purity confirmed generally as ≥99%[3] for single substance or non-mixture evaluations (see NTP 2006 for example of study effects attributable to impurities of approximately 1%),

AND that exposure was consistently administered (i.e., with the same method and time-frame) across treatment groups.

Co, CaCo, CrSe, CaS: There is direct evidence that exposure was consistently assessed (i.e., under the same method and time-frame) using well-established methods that directly measure exposure (e.g., measurement of the chemical in air or measurement of the chemical in blood, plasma, urine, etc.),

OR exposure was assessed using less-established methods that directly measure exposure and are validated against well-established methods.

Probably Low risk of bias:

HCT, EA: There is indirect evidence that the exposure (including purity and stability of the test substance and compliance with the treatment, if applicable) was independently characterized and purity confirmed generally as ≥99%[3] (i.e., the supplier of the chemical provides documentation of the purity of the chemical),

OR direct evidence that purity was independently confirmed as ≥98%[3] it is deemed that impurities of up to 2% would not appreciably bias results,

AND there is indirect evidence that exposure was consistently administered (i.e., with the same method and time-frame) across treatment groups.

Co, CaCo, CrSe, CaS: There is indirect evidence that the exposure was consistently assessed using well-established methods that directly measure exposure,

OR exposure was assessed using indirect measures (e.g., questionnaire or occupational exposure assessment by a certified industrial hygienist) that have been validated or empirically shown to be consistent with methods that directly measure exposure (i.e., inter-methods validation: one method vs. another).

Probably High risk of bias:

HCT, EA: There is indirect evidence that the exposure (including purity and stability of the test substance and compliance with the treatment, if applicable) was assessed using poorly validated methods,

OR there is insufficient information provided about the validity of the exposure assessment method, but no evidence for concern (record “NR” as basis for answer).

---

[3] Note purity thresholds should be developed for specific research questions and reflect empirical data for the substance and outcome under consideration when possible. Therefore, the appropriate cut-off purity value may be lower or higher than the values listed below for ≥99% defining the difference between “definitely low” and “probably low” or ≥98% defining the difference between “probably low” and “probably high” risk of bias.

Co, CaCo, CrSe, CaS: There is indirect evidence that the exposure was assessed using poorly validated methods that directly measure exposure,

OR there is direct evidence that the exposure was assessed using indirect measures that have not been validated or empirically shown to be consistent with methods that directly measure exposure (e.g., a job-exposure matrix or self-report without validation) (record "NR" as basis for answer),

OR there is insufficient information provided about the exposure assessment, including validity and reliability, but no evidence for concern about the method used (record "NR" as basis for answer).

Definitely High risk of bias:

HCT, EA: There is direct evidence that the exposure (including purity and stability of the test substance and compliance with the treatment, if applicable) was assessed using poorly validated methods.

Co, CaCo, CrSe, CaS: There is direct evidence that the exposure was assessed using methods with poor validity,

OR evidence of exposure misclassification (e.g., differential recall of self-reported exposure).

9. Can we be confident in the outcome assessment?

Confidence in the outcome requires valid, reliable, and sensitive methods to assess the outcome applied consistently across groups. Outcome misclassification or measurement error may be unrelated to the exposure (non-differential) or related to the exposure (differential). Non-differential measurement error of outcomes will usually bias the results toward the null by lowering precision and therefore reducing the ability to distinguish potential effects on exposure between exposure levels. Differential measurement error of outcomes can bias the exposure-outcome relationship and result in detection bias. There are three important factors for assessing bias in the outcome assessment: 1) the objectivity of the outcome assessment, 2) consistency in measurement of outcomes, and 3) blinding of the outcome assessors (for knowledge of the exposure).

Detection bias can be minimized by using valid and reliable methods to assess the outcome applied consistently across groups (i.e., under the same method and time-frame). Objectivity of the outcome assessment and the need for blinding are two sides of the same issue. Blinding requires that outcome assessors do not know the study group or exposure level of the human subject or animal when the outcome was assessed. The objectivity of procedures used for measuring and reporting an outcome will impact the degree to which outcome assessors could bias the reported results. For example, a behavioral outcome rated by a researcher (i.e., direct observation of behaviors) relies on subjective judgment and therefore may be impacted by potential bias of the outcome assessor to a greater degree than outcomes that are measured by machines (e.g., automated red blood cell counts). Similarly, studies relying on self-report of outcome may be rated as having a higher risk of bias than studies with clinically observed outcomes (Viswanathan *et al.* 2012). Although objective measures are less prone to bias by researchers than subjective measures, bias could be introduced during sample preparation or handling and therefore blinding still has a role in controlling for potential bias unless sample preparation and outcome measurement are accomplished with automated procedures. For example, the potential for outcome assessors to introduce bias would be minimized for *ex vivo* studies where samples are collected and outcomes are assessed automatically within an apparatus.

Acceptable methods for measuring the outcomes of interest will be highly dependent on the outcome and therefore a specific list of acceptable, inaccurate, or potentially biased methods should be

developed for each evaluation and will require subject-matter expertise. It is recommended that experts with some knowledge of the literature (including both exposure and outcome) participate in drafting the risk-of-bias criteria for outcome assessment when a review protocol is developed. Even with early expert consultation and planning, outcome questions may arise when the actual studies are assessed because of non-traditional methods, application to non-traditional species, or endpoints that are indirectly related to the outcome of interest. Additional consultation and modifications to the outcome risk-of-bias criteria may be necessary. When changes are made, they should be documented along with the date on which modifications were made and the logic for the changes.

Human introductory text: Differential methods used in the assessment of outcomes is a source of bias and this is a widely used risk-of-bias element in tools for observational human studies (Downs and Black 1998, Genaidy *et al.* 2007, Shamliyan *et al.* 2010, Viswanathan *et al.* 2012, Sterne *et al.* 2014). The recent guidance for non-randomized studies of interventions suggests considering the objectivity of the outcome assessment when evaluating bias in the outcome assessment (Sterne *et al.* 2014) and we have included consideration of the objectivity in this document for evaluating the potential impact of blinding practices. Blinding of outcome assessors is a widely recommended risk-of-bias element for controlled trials and observational studies (Higgins and Green 2011, Viswanathan *et al.* 2012, Sterne *et al.* 2014). For human studies blinding of the subject to exposure levels should also be considered. For example, a subject's knowledge of their own exposure levels would represent an increased risk of bias for self-reported outcomes relative to clinically measured outcomes.

Without distinguishing between the different stages of blinding during the conduct of a study, lack of blinding in randomized trials has been empirically shown to be associated with larger estimations of intervention effects (on average a 9% increase in an odds ratio) (Pildal *et al.* 2007). Schulz et al. (1995) analyzed 250 controlled trials and found that studies that were not double-blinded had a 17% larger estimation of treatment effect, on average. In trials with more subjective outcomes, more bias has been observed with lack of blinding (Wood *et al.* 2008), indicating that blinding outcome assessors could be more important for these effects.

For some exposures, it is not possible to entirely blind outcome assessors, particularly if subjects are self-reporting outcomes. In practice, successful blinding cannot always be ensured, as it can be compromised for most interventions. In some cases the treatment may have side effects possibly allowing the participant to detect which intervention they received, unless the study compares interventions with similar side effects or uses an active placebo (Boutron *et al.* 2006).

Animal introductory text: Blinding of outcome assessors is a widely recommended risk-of-bias element for animal studies (reviewed in Krauth *et al.* 2013). This tool assesses blinding and also considers differential methods, procedures, or time points for measuring outcomes to be a source of bias based on the common use of this element in study quality tools for human controlled trials and observational studies (Higgins and Green 2011, Viswanathan *et al.* 2012, Sterne *et al.* 2014).

There is empirical evidence that lack of blinding at outcome assessment in animal studies is associated with larger measures of the effect (Bebarta *et al.* 2003, Sena *et al.* 2007, Vesterinen *et al.* 2010). In animal studies, blinding of study group at outcome assessment may not be possible because of the need to determine if treated animals are affected relative to controls in a treatment or dose-dependent manner (examples include clinical observations and histopathological assessment of non-neoplastic lesions). However, animal studies are in general more tightly controlled than human studies and additional measures may be taken to reduce the risk of bias.

Note: for case-control studies, confirmation that the control subjects are free of the outcome is considered under as separate risk-of-bias question, "Did selection of study participants result in appropriate comparison groups?"

There are separate risk-of-bias issues affected by concealment or blinding that are important for selection, performance and detection bias: 1) a question under selection bias addresses allocation concealment of research personnel and human subjects; and 2) a question under performance bias addresses blinding of research personnel and human subjects to the study group during the study.

Previous versions of the OHAT risk-of-bias tool had separate questions for "Can we be confident in the outcome assessment" and "Were the outcome assessors blinded to study group or exposure level?" These two questions are interrelated and therefore have been combined as factors to consider for a single risk-of-bias rating on confidence in the outcome assessment. Recent guidance, such as the Cochrane risk-of-bias tool for non-randomized studies of intervention (Sterne et al. 2014) has taken a similar approach for addressing blinding and outcome measurement in a single question on outcome assessment.

Applies to: HCT, EA, Co, CaCo, CrSe, CaS

Definitely Low risk of bias:

HCT, Co: There is direct evidence that the outcome was assessed using well-established methods (e.g., the "gold standard" with validity and reliability >0.70 Genaidy *et al.* 2007),

AND subjects had been followed for the same length of time in all study groups. Acceptable assessment methods will depend on the outcome, but examples of such methods may include: objectively measured with diagnostic methods, measured by trained interviewers, obtained from registries (Shamliyan *et al.* 2010),

AND there is direct evidence that the outcome assessors (including study subjects, if outcomes were self-reported) were adequately blinded to the study group, and it is unlikely that they could have broken the blinding prior to reporting outcomes.

EA: There is direct evidence that the outcome was assessed using well-established methods (the gold standard),

AND assessed at the same length of time after initial exposure in all study groups,

AND there is direct evidence that the outcome assessors were adequately blinded to the study group, and it is unlikely that they could have broken the blinding prior to reporting outcomes.

CaCo: There is direct evidence that the outcome was assessed in cases (i.e., case definition) and controls using well-established methods (the gold standard),

AND subjects had been followed for the same length of time in all study groups,

AND there is direct evidence that the outcome assessors (including study subjects, if outcomes were self-reported) were adequately blinded to the exposure level when outcome was assessed in cases (i.e., case definition) and controls.

CrSe, CaS: There is direct evidence that the outcome was assessed using well-established methods (the gold standard),

AND there is direct evidence that the outcome assessors (including study subjects, if outcomes were self-reported) were adequately blinded to the exposure level, and it is unlikely that they could have broken the blinding prior to reporting outcomes.

Probably Low risk of bias:

HCT, Co: There is indirect evidence that the outcome was assessed using acceptable methods (i.e., deemed valid and reliable but not the gold standard) (e.g., validity and reliability ≥0.40 Genaidy *et al.* 2007),

AND subjects had been followed for the same length of time in all study groups [Acceptable, but not ideal assessment methods will depend on the outcome, but examples of such methods may include proxy reporting of outcomes and mining of data collected for other purposes],
OR it is deemed that the outcome assessment methods used would not appreciably bias results,
AND there is indirect evidence that the outcome assessors (including study subjects, if outcomes were self-reported) were adequately blinded to the study group, and it is unlikely that they could have broken the blinding prior to reporting outcomes,
OR it is deemed that lack of adequate blinding of outcome assessors would not appreciably bias results, which is more likely to apply to objective outcome measures.

EA: There is indirect evidence that the outcome was assessed using acceptable methods (i.e., deemed valid and reliable but not the gold standard),
AND assessed at the same length of time after initial exposure in all study groups,
OR it is deemed that the outcome assessment methods used would not appreciably bias results,
AND there is indirect evidence that the outcome assessors were adequately blinded to the study group, and it is unlikely that they could have broken the blinding prior to reporting outcomes,
OR it is deemed that lack of adequate blinding of outcome assessors would not appreciably bias results, which is more likely to apply to objective outcome measures. For some outcomes, particularly histopathology assessment, outcome assessors are not blind to study group as they require comparison to the control to appropriately judge the outcome, but additional measures such as multiple levels of independent review by trained pathologists can minimize this potential bias.

CaCo: There is indirect evidence that the outcome was assessed in cases (i.e., case definition) and controls using acceptable methods,
AND subjects had been followed for the same length of time in all study groups,
OR it is deemed that the outcome assessment methods used would not appreciably bias results,
AND there is direct evidence that the outcome assessors were adequately blinded to the exposure level when reporting outcomes,
OR it is deemed that lack of adequate blinding of outcome assessors would not appreciably bias results (including that subjects self-reporting outcomes were likely not aware of reported links between the exposure and outcome or lack of blinding is unlikely to bias a particular outcome).

CrSe, CaS: There is indirect evidence that the outcome was assessed using acceptable methods,
OR it is deemed that the outcome assessment methods used would not appreciably bias results,
AND there is indirect evidence that the outcome assessors were adequately blinded to the exposure level, and it is unlikely that they could have broken the blinding prior to reporting outcomes,
OR it is deemed that lack of adequate blinding of outcome assessors would not appreciably bias results (including that subjects self-reporting outcomes were likely not aware of reported links between the exposure and outcome lack of blinding is unlikely to bias a particular outcome).

Probably High risk of bias:

HCT, Co: There is indirect evidence that the outcome assessment method is an insensitive instrument (e.g., a questionnaire used to assess outcomes with no information on validation),
OR the length of follow up differed by study group,
OR there is indirect evidence that it was possible for outcome assessors (including study subjects if outcomes were self-reported) to infer the study group prior to reporting outcomes,
OR there is insufficient information provided about blinding of outcome assessors (record "NR" as basis for answer).

EA: There is indirect evidence that the outcome assessment method is an insensitive instrument,

OR the length of time after initial exposure differed by study group,

OR there is indirect evidence that it was possible for outcome assessors to infer the study group prior to reporting outcomes without sufficient quality control measures,

OR there is insufficient information provided about blinding of outcome assessors (record "NR" as basis for answer).

CaCo: There is indirect evidence that the outcome was assessed in cases (i.e., case definition) using an insensitive instrument,

OR there is insufficient information provided about how cases were identified (record "NR" as basis for answer),

OR there is indirect evidence that it was possible for outcome assessors to infer the exposure level prior to reporting outcomes (including that subjects self-reporting outcomes were likely aware of reported links between the exposure and outcome),

OR there is insufficient information provided about blinding of outcome assessors (record "NR" as basis for answer).

CrSe, CaS: There is indirect evidence that the outcome assessment method is an insensitive instrument,

OR there is indirect evidence that it was possible for outcome assessors to infer the exposure level prior to reporting outcomes (including that subjects self-reporting outcomes were likely aware of reported links between the exposure and outcome),

OR there is insufficient information provided about blinding of outcome assessors (record "NR" as basis for answer).

Definitely High risk of bias:

HCT, Co: There is direct evidence that the outcome assessment method is an insensitive instrument,

OR the length of follow up differed by study group,

OR there is direct evidence for lack of adequate blinding of outcome assessors (including study subjects if outcomes were self-reported), including no blinding or incomplete blinding.

EA: There is direct evidence that the outcome assessment method is an insensitive instrument,

OR the length of time after initial exposure differed by study group,

OR there is direct evidence for lack of adequate blinding of outcome assessors, including no blinding or incomplete blinding without quality control measures.

CaCo: There is direct evidence that the outcome was assessed in cases (i.e., case definition) using an insensitive instrument,

OR there is direct evidence that outcome assessors were aware of the exposure level prior to reporting outcomes (including that subjects self-reporting outcomes were aware of reported links between the exposure and outcome).

CrSe, CaS: There is direct evidence that the outcome assessment method is an insensitive instrument,

OR there is direct evidence that outcome assessors were aware of the exposure level prior to reporting outcomes (including that subjects self-reporting outcomes were aware of reported links between the exposure and outcome).

Selective Reporting Bias

Selective reporting bias refers to selective inclusion of outcomes in the publication of the study on the basis of the results (Hutton and Williamson 2000, Higgins and Green 2011).

10. Were all measured outcomes reported?

Selective reporting of results is a recommended element of assessing risk of bias (Guyatt *et al.* 2011, Higgins *et al.* 2011, IOM 2011, Viswanathan *et al.* 2012). Selective reporting is present if pre-specified outcomes are not reported or incompletely reported. It is likely widespread and difficult to assess with confidence for most studies unless the study protocol is available. Selective reporting bias can be assessed by comparing the “methods” and “results” section of the paper, and by considering outcomes measured in the context of knowledge in the field. Abstracts of presentations relating to the study may contain information about outcomes not subsequently mentioned in publications. Selective reporting bias should be suspected if the study does not report outcomes in the results section that would have been expected based on the methods, or if a composite score is present without the individual component outcomes (Guyatt *et al.* 2011). It may be useful to pay attention to author affiliations and funding source which can contribute to selective outcome reporting when results are not consistent with expectations or value to the research objectives.

Applies to: HCT, EA, Co, CaCo, CrSe, CaS

Definitely Low risk of bias:

HCT, EA, Co, CaCo, CrSe, CaS: There is direct evidence that all of the study’s measured outcomes (primary and secondary) outlined in the protocol, methods, abstract, and/or introduction (that are relevant for the evaluation) have been reported. This would include outcomes reported with sufficient detail to be included in meta-analysis or fully tabulated during data extraction and analyses had been planned in advance.

Probably Low risk of bias:

HCT, EA, Co, CaCo, CrSe, CaS: There is indirect evidence that all of the study’s measured outcomes (primary and secondary) outlined in the protocol, methods, abstract, and/or introduction (that are relevant for the evaluation) have been reported,

OR analyses that had not been planned in advance (i.e., retrospective unplanned subgroup analyses) are clearly indicated as such and it is deemed that the unplanned analyses were appropriate and selective reporting would not appreciably bias results (e.g., appropriate analyses of an unexpected effect). This would include outcomes reported with insufficient detail such as only reporting that results were statistically significant (or not).

Probably High risk of bias:

HCT, EA, Co, CaCo, CrSe, CaS: There is indirect evidence that all of the study’s measured outcomes (primary and secondary) outlined in the protocol, methods, abstract, and/or introduction (that are relevant for the evaluation) have been reported,

OR and there is indirect evidence that unplanned analyses were included that may appreciably bias results,

OR there is insufficient information provided about selective outcome reporting (record "NR" as basis for answer).

Definitely High risk of bias:

HCT, EA, Co, CaCo, CrSe, CaS: There is direct evidence that all of the study's measured outcomes (primary and secondary) outlined in the protocol, methods, abstract, and/or introduction (that are relevant for the evaluation) have not been reported. In addition to not reporting outcomes, this would include reporting outcomes based on composite score without individual outcome components or outcomes reported using measurements, analysis methods or subsets of the data (e.g., subscales) that were not pre-specified or reporting outcomes not pre-specified, or that unplanned analyses were included that would appreciably bias results.

Other Bias

11. Were there no other potential threats to internal validity (e.g., statistical methods were appropriate and researchers adhered to the study protocol)?

On a project specific basis, additional questions for other potential threats to internal validity can be added and applied to study designs as appropriate.

Were statistical methods appropriate?

Some of the more extensive quality tools have a separate question for appropriateness of the statistical methods (e.g., 1 of the 25 elements in the Downs and Black 1998 tool addresses the statistics); however most do not include a separate question. The OHAT risk-of-bias tool suggests consideration of statistical methods with the other potential threats to internal validity. One of the common statistical issues identified has been reporting of statistical tests that require normally distributed data (e.g., t-test or ANOVA) without reporting that the homogeneity of variance was tested or confirmed.

It is recommended that experts with some knowledge of statistical methods used in the literature participate in drafting the risk-of-bias criteria for identifying inappropriate statistical methods when a review protocol is developed. Even with early expert consultation and planning, statistical methods questions may arise when the actual studies are assessed. Additional consultation and modifications to the statistical methods risk-of-bias criteria may be necessary. When changes are made, they should be documented along with the date on which modifications were made and the logic for the changes.

Did researchers adhere to the study protocol?

Failure of the study to maintain fidelity to the protocol is recommended as an important consideration when assessing performance bias (IOM 2011, Viswanathan *et al.* 2012). However, it will likely be difficult to assess with confidence for most studies, particularly when the methods section of a publication is all that is available. In some instances the protocol is meant to be "fluid" and the protocol explicitly allows for modification based on need; such fluidity does not mean the interventions are implemented incorrectly. The deviation may not result in a risk of bias, or if it does the direction of the bias (towards or away from the null) will differ based on the deviation from the protocol.

We recognize that given reporting practices it is unlikely that deviations from the protocol will be explicitly reported in most studies. Thus, we will assume unless stated otherwise that no deviations occurred which will result in most studies considered “probably low risk of bias”. In the short-term, this risk-of-bias element is unlikely to be informative for the purposes of discriminating between studies of higher quality and studies of lower quality. However, in the long-term, especially if reporting standards improve, collecting this information may generate data that will allow us to empirically assess evidence of bias or to remove this risk-of-bias question from consideration.

Animal introductory text: One of the more common deviations from protocol that can occur in toxicity studies is when a dose level is decreased based on evidence of mortality or severe toxicity. However, depending upon how the author addresses this change it may or may not impact results. For example, when this occurs in NTP studies, the usual analysis would be conducted on the dose groups remaining after the toxic dose level is dropped. A similar situation arises when a dose group has to be euthanized due to overt toxicity.

Previous versions of the OHAT risk-of-bias tool had a separate question for “Did researchers adhere to the study protocol?” The overwhelming majority of studies examined during case study evaluations were not reported in sufficient detail to permit a meaningful answer to whether or not the study adhered to a study protocol. Therefore, we will continue to collect data on this element, but have moved it to be considered under other potential threats to internal validity.

Did the study design or analysis account for important confounding and modifying variables (including unintended co-exposures) in experimental studies?

There is a separate risk-of-bias question to address confounding and modifying variables (including co-exposures) for observational studies because confounding is a much greater concern for observational studies. Controlled exposure studies (i.e., experimental human or animal studies) can address confounding through study design features such as randomization and allocation concealment. Therefore, most study quality tools for experimental studies do not include questions for confounding (Higgins *et al.* 2011, Krauth *et al.* 2013, Koustas *et al.* 2014). Confounding by chance (i.e., confounding that is unknown, unmeasured, or poorly measured) is expected to be equally distributed between groups under true randomization; however, experimental studies may not always successfully randomize potential confounders (Viswanathan *et al.* 2013). Recognizing this, the SYRCLE risk-of-bias tool for experimental animal studies asks whether groups were “similar at baseline or were they adjusted for confounders in the analysis”(Hooijmans *et al.* 2014). In the context of an animal study, this element would include consideration of covariates such as body weight, litter size, or other outcome-specific covariates. Similarly, the 2012 risk-of-bias guidance from AHRQ recommends consideration of confounding for randomized clinical trials. For this tool, we have only included the consideration of confounding in controlled exposure studies (i.e., experimental human or animal studies) under “other potential threats to internal validity” for cases where it is strongly suspected because randomization and allocation concealment should address the issue of confounding. The issue of confounding overlaps with randomization and allocation concealment, and multiple questions would address the same issue.

Animal introductory text: Randomization and allocation concealment in experimental studies address should result in non-differential distribution of potential confounders or co-exposures. Experimental study design generally reduces or eliminates co-exposures; however the impact of inadvertent chemical or biological co-exposures should be considered. For example, if the experimental exposure is to bisphenol A or other chemical with estrogenic properties, husbandry practices that raise the background level of estrogenicity across all study groups (e.g., a diet high in phytoestrogens) may make the model

system less sensitive to detect low-dose effects of BPA (Thigpen *et al.* 2007, Muhlhauser *et al.* 2009). In this case, the direction of the bias would be towards the null (towards smaller effect sizes). Infectious agents and non-treatment related co-morbidity should also be monitored as potential sources of bias.

The direction of the bias will depend on the nature of co-exposure and whether or not there are differences between study groups. For example, certain types of infections may be related to outcomes of interest (reviewed in NRC 1991, Baker 1998, GV-SOLAS 1999). *Helicobacter pylori* is a bacterial carcinogen and may cause may cause chronic active hepatitis, hepatic tumors, and proliferative typhlocolitis in rodents (Kusters *et al.* 2006). If the infection occurs in control animals or across all study groups, then the bias for an effect on the liver may be towards the null (smaller effect size). If the infection occurs only in treated animals, then the bias for an effect on the liver may be away from the null (larger effect size).

Examples:

- **Statistics:** Failure to statistically or experimentally adjust for litter in an animal study with a developmental outcome. The direction of the bias is away from the null towards a larger effect size (Haseman *et al.* 2001).
- **Deviations from the protocol:** Evidence of deviations in the protocol should be noted as direct (definitely high risk of bias) or indirect (probably high risk of bias). Given reporting practices it is unlikely that deviations from the protocol will be explicitly reported in most studies and therefore the bias is very difficult to assess. Caution should be taken so that studies that do provide a protocol and report deviations are not ”punished” for having better reporting practices.
- **Unintended co-exposures for experimental studies**: Evidence of other exposures that are anticipated to bias results should be noted as direct (definitely high risk of bias) or indirect (probably high risk of bias) evidence of other exposures anticipated to bias results, if present and not appropriately adjusted for. Non-differential co-exposures that are likely to bias the results toward the null should be considered in the context of the study findings.

REFERENCES

Baker DG. 1998. Natural pathogens of laboratory mice, rats, and rabbits and their effects on research. *Clin Microbiol Rev* 11(2): 231-266.

Bebarta V, Luyten D, Heard K. 2003. Emergency medicine animal research: does use of randomization and blinding affect the results? *Acad Emerg Med* 10(6): 684-687.

Boutron I, Estellat C, Guittet L, Dechartres A, Sackett DL, Hrobjartsson A, Ravaud P. 2006. Methods of blinding in reports of randomized controlled trials assessing pharmacologic treatments: a systematic review. *PLoS Med* 3(10): e425.

CLARITY Group at McMaster University. 2013. *Tools to assess risk of bias in cohort studies, case control studies, randomized controlled trials, and longitudinal symptom research studies aimed at the general population*. Available: http://www.evidencepartners.com/resources/ [accessed 15 January 2013].

Downs SH, Black N. 1998. The feasibility of creating a checklist for the assessment of the methodological quality both of randomised and non-randomised studies of health care interventions. *J Epidemiol Community Health* 52(6): 377-384.

Duke JL, Zammit TG, Lawson DM. 2001. The effects of routine cage-changing on cardiovascular and behavioral parameters in male Sprague-Dawley rats. *Contemp Top Lab Anim Sci* 40(1): 17-20.

Durda J, Preziosi D. 2000. Data quality evaluation of toxicological studies used to derive ecotoxicological benchmarks. *Human and Ecological Risk Assessment: An International Journal* 6(5): 747-765.

Dwan K, Gamble C, Kolamunnage-Dona R, Mohammed S, Powell C, Williamson PR. 2010. Assessing the potential for outcome reporting bias in a review: a tutorial. *Trials* 11: 52.

Genaidy AM, Lemasters GK, Lockey J, Succop P, Deddens J, Sobeih T, Dunning K. 2007. An epidemiological appraisal instrument - a tool for evaluation of epidemiological studies. *Ergonomics* 50(6): 920-960.

Gerdin AK, Igosheva N, Roberson LA, Ismail O, Karp N, Sanderson M, Cambridge E, Shannon C, Sunter D, Ramirez-Solis R, Bussell J, White JK. 2012. Experimental and husbandry procedures as potential modifiers of the results of phenotyping tests. *Physiol Behav* 106(5): 602-611.

Gerstman BB. 2013. *Epidemiology kept simple* 3rd ed., New York, NY: Wiley-Blackwell.

Guyatt GH, Oxman AD, Vist G, Kunz R, Brozek J, Alonso-Coello P, Montori V, Akl EA, Djulbegovic B, Falck-Ytter Y, Norris SL, Williams JW, Jr., Atkins D, Meerpohl J, Schunemann HJ. 2011. GRADE guidelines: 4. Rating the quality of evidence-study limitations (risk of bias). *J Clin Epidemiol* 64(4): 407-415.

GV-SOLAS. 1999. Implications of infectious agents on results of animal experiments. Report of the Working Group on Hygiene of the Gesellschaft fur Versuchstierkunde--Society for Laboratory Animal Science (GV-SOLAS). *Lab Anim* 33 Suppl 1: S39-87.

Haseman JK, Bailer AJ, Kodell RL, Morris R, Portier K. 2001. Statistical issues in the analysis of low-dose endocrine disruptor data. *Toxicol Sci* 61(2): 201-210.

Higgins J, Green S. 2011. *Cochrane Handbook for Systematic Reviews of Interventions*. The Cochrane Collaboration. Available: www.cochrane-handbook.org [accessed 3 February 2013].

Higgins JP, Altman DG, Gotzsche PC, Juni P, Moher D, Oxman AD, Savovic J, Schulz KF, Weeks L, Sterne JA. 2011. The Cochrane Collaboration's tool for assessing risk of bias in randomised trials. *Br Med J* 343: d5928.

Hooijmans CR, Rovers MM, de Vries RB, Leenaars M, Ritskes-Hoitinga M, Langendam MW. 2014. SYRCLE's risk of bias tool for animal studies. *BMC medical research methodology* 14(1): 43.

Hutton JL, Williamson PR. 2000. Bias in meta-analysis due to outcome variable selection within studies. *Journal of the Royal Statistical Society Series C-Applied Statistics* 49: 359-370.

IOM (Institute of Medicine). 2011. *Finding what works in health care: Standards for systematic reviews*. Washington, DC, The National Academies Press: 318. Available: http://www.nap.edu/openbook.php?record_id=13059 [accessed 3 May 2013].

Johnson P, Sutton P, Atchley D, Koustas E, Lam J, Robinson K, Sen S, Axelrad D, Woodruff T. 2013. *Applying the Navigation Guide: Case study #1. The impact of developmental exposure to perfluorooctanoic acid (PFOA) on fetal growth. A systematic review of the human evidence - Protocol*. Available: http://prhe.ucsf.edu/prhe/pdfs/PFOA%20Human%20Protocol.pdf.

Johnson PI, Sutton P, Atchley DS, Koustas E, Lam J, Sen S, Robinson KA, Axelrad DA, Woodruff TJ. 2014. The Navigation Guide - evidence-based medicine meets environmental health: systematic review of human evidence for PFOA effects on fetal growth. *Environ Health Perspect* 122(10): 1028-1039.

Koustas E, Lam J, Sutton P, Johnson P, Atchley D, Robinson K, Sen S, Axelrad D, Woodruff T. 2013. *Applying the Navigation Guide: Case Study #1. The Impact of Developmental Exposure to Perfluorooctanoic Acid (PFOA) On Fetal Growth. A Systematic Review of the Non-Human Evidence - Protocol*. Available: http://prhe.ucsf.edu/prhe/pdfs/PFOA%20NON-HUMAN%20PROTOCOL.pdf.

Koustas E, Lam J, Sutton P, Johnson PI, Atchley DS, Sen S, Robinson KA, Axelrad DA, Woodruff TJ. 2014. The Navigation Guide - evidence-based medicine meets environmental health: systematic review of nonhuman evidence for PFOA effects on fetal growth. *Environ Health Perspect* 122(10): 1015-1027.

Krauth D, Woodrull T, Bero L. 2013. Instruments for assessing risk of bias and other methodological criteria of published animal studies: a systematic review. *Environ Health Perspect* 121(9): 985-992.

Kusters JG, van Vliet AH, Kuipers EJ. 2006. Pathogenesis of Helicobacter pylori infection. *Clin Microbiol Rev* 19(3): 449-490.

Macleod MR, van der Worp HB, Sena ES, Howells DW, Dirnagl U, Donnan GA. 2008. Evidence for the efficacy of NXY-059 in experimental focal cerebral ischaemia is confounded by study quality. *Stroke* 39(10): 2824-2829.

Muhlhauser A, Susiarjo M, Rubio C, Griswold J, Gorence G, Hassold T, Hunt PA. 2009. Bisphenol A effects on the growing mouse oocyte are influenced by diet. *Biol Reprod* 80(5): 1066-1071.

NRC (National Research Council,). 1991. *Infectious diseases of mice and rats*. 9780309063326. Washington, DC, Press TNA: 397. Available: http://www.nap.edu/openbook.php?record_id=1429.

NTP (National Toxicology Program,). 2006. *Toxicology and carcinogenesis studies of a binary mixture of 3,3',4,4',5-Pentachlorobiphenyl (PCB 126) (CAS No. 57465-28-8) and 2,3',4,4',5-Pentachlorobiphenyl (PCB 118) (CAS No. 31508-00-6) in female Harlan Sprague-Dawley rats (Gavage Studies)*. Available: http://ntp.niehs.nih.gov/?objectid=D16D6C59-F1F6-975E-7D23D1519B8CD7A5 [accessed 28 January 2013].

NTP (National Toxicology Program). 2013a. *Informational meeting on the draft OHAT Approach for Systematic Review and Evidence Integration for Literature-based Health Assessments. April 23, 2013*. Available: http://ntp.niehs.nih.gov/go/38751 [accessed 28 January 2014].

NTP (National Toxicology Program). 2013b. *Draft Protocol for Systematic Review to Evaluate the Evidence for an Association Between Bisphenol A (BPA) and Obesity*. RTP, NC: Office of Health Assessment and Translation. Available: http://ntp.niehs.nih.gov/go/38673 [accessed 9 April 2013].

NTP (National Toxicology Program). 2013c. *Draft Protocol for Systematic Review to Evaluate the Evidence for an Association Between Perfluorooctanoic Acid (PFOA) or Perfluorooctane Sulfonate (PFOS) Exposure and Immunotoxicity*. RTP, NC: Office of Health Assessment and Translation. Available: http://ntp.niehs.nih.gov/go/38673 [accessed 9 April 2013].

NTP (National Toxicology Program). 2013d. *Draft OHAT Approach for Systematic Review and Evidence Integration for Literature-based Health Assessments – February 2013*. RTP, NC: Office of Health Assessment and Translation. Available: http://ntp.niehs.nih.gov/go/38138 [accessed 10 March 2013].

Pildal J, Hrobjartsson A, Jorgensen KJ, Hilden J, Altman DG, Gotzsche PC. 2007. Impact of allocation concealment on conclusions drawn from meta-analyses of randomized trials. *Int J Epidemiol* 36(4): 847-857.

Rosenthal R, Lawson R. 1964. A Longitudinal Study of the Effects of Experimenter Bias on the Operant Learning of Laboratory Rats. *Journal of psychiatric research* 69: 61-72.

Rothman KJ, Greenland S, Lash TL. 2012. *Modern Epidemiology* 3rd ed., Boston, MA: Lippincott, Williams & Wilkins.

Schulz KF, Chalmers I, Hayes RJ, Altman DG. 1995. Empirical evidence of bias. Dimensions of methodological quality associated with estimates of treatment effects in controlled trials. *J Am Med Assoc* 273(5): 408-412.

Schulz KF, Altman DG, Moher D. 2002. Allocation concealment in clinical trials. *J Am Med Assoc* 288(19): 2406-2407; author reply 2408-2409.

Sena E, van der Worp HB, Howells D, Macleod M. 2007. How can we improve the pre-clinical development of drugs for stroke? *TRENDS Neurosci* 30(9): 433-439.

Shamliyan T, Kane RL, Dickinson S. 2010. A systematic review of tools used to assess the quality of observational studies that examine incidence or prevalence and risk factors for diseases. *J Clin Epidemiol* 63(10): 1061-1070.

Shamliyan TA, Kane RL, Ansari MT, Raman G, Berkman ND, Grant M, Janes G, Maglione M, Moher D, Nasser M, Robinson KA, Segal JB, Tsouros S. 2011. Development of quality criteria to evaluate nontherapeutic studies of incidence, prevalence or risk factors of chronic diseases: Pilot study of new checklists. Available at http://www.ncbi.nlm.nih.gov/books/NBK53272/ [accessed March 6, 2012]. *Rockville (MD): Agency for Healthcare Research and Quality (US); 2011 Jan. Report No.: 11-EHC008-EF. AHRQ Methods for Effective Health Care.*

Sterne J, Higgins J, Reeves B, on behalf of the development group for ACROBAT-NRSI. 2014. *A Cochrane Risk Of Bias Assessment Tool: for Non-Randomized Studies of Interventions (ACROBAT-NRSI), Version 1.0.0*. Available: http://www.riskofbias.info [accessed 28 September 2014].

Szklo M, Nieto FJ. 2007. *Epidemiology: Beyond the Basics* 2nd ed., Sudbury, MA: Jones and Bartlett Publishers.

Thigpen JE, Setchell KD, Padilla-Banks E, Haseman JK, Saunders HE, Caviness GF, Kissling GE, Grant MG, Forsythe DB. 2007. Variations in phytoestrogen content between different mill dates of the same diet produces significant differences in the time of vaginal opening in CD-1 mice and F344 rats but not in CD Sprague-Dawley rats. *Environ Health Perspect* 115(12): 1717-1726.

Vesterinen HM, Sena ES, ffrench-Constant C, Williams A, Chandran S, Macleod MR. 2010. Improving the translational hit of experimental treatments in multiple sclerosis. *Mult Scler* 16(9): 1044-1055.

Viswanathan M, Ansari M, Berkman ND, Chang S, Hartling L, McPheeters LM, Santaguida PL, Shamliyan T, Singh K, Tsertsvadze A, Treadwell JR. 2012. *Assessing the risk of bias of individual studies when comparing medical interventions*. Publication No. 12-EHC047-EF. Rockville, MD. Agency for Healthcare Research and Quality (AHRQ). Available:

http://www.effectivehealthcare.ahrq.gov/index.cfm/search-for-guides-reviews-and-reports/?pageaction=displayproduct&productid=998 [accessed 3 January 2013].

Viswanathan M, Ansari M, Berkman ND, Chang S, Hartling L, McPheeters LM, Santaguida PL, Shamliyan T, Singh K, Tsertsvadze A, Treadwell JR. 2013. *Assessing risk of bias and confounding in observational studies of interventions or exposures: Further development of the RTI item bank*. Publication No. 13-EHC106-EF. Rockville, MD. Agency for Healthcare Research and Quality (AHRQ). Available: http://www.effectivehealthcare.ahrq.gov/ehc/products/414/1612/RTI-item-bank-bias-precision-130805.pdf [accessed 11 January 2014].

Wells G, Shea B, O'Connell D, Peterson J, Welch V, Losos M, Tugwell P. 2014. *The Newcastle-Ottawa Scale (NOS) for assessing the quality of nonrandomised studies in meta-analyses*. Ottawa Hospital Research Institute. Available: http://www.ohri.ca/programs/clinical_epidemiology/oxford.asp [accessed 20 February 2014].

White E. 2003. Design and interpretation of studies of differential exposure measurement error. *Am J Epidemiol* 157(5): 380-387.

Wood L, Egger M, Gluud LL, Schulz KF, Juni P, Altman DG, Gluud C, Martin RM, Wood AJ, Sterne JA. 2008. Empirical evidence of bias in treatment effect estimates in controlled trials with different interventions and outcomes: meta-epidemiological study. *Br Med J* 336(7644): 601-605.

Woodruff TJ, Sutton P. 2014. The Navigation Guide systematic review methodology: a rigorous and transparent method for translating environmental health science into better health outcomes. *Environ Health Perspect* 122(10): 1007-1014.
